# Prevalence of Actionable Pharmacogenetic Genotype Frequencies, Cautionary Medication Use, and Polypharmacy in Community‐Dwelling Older Adults

**DOI:** 10.1002/cpt.3702

**Published:** 2025-04-30

**Authors:** Chad A. Bousman, Ankita Narang, Ziad Al Bkhetan, Robyn L. Woods, Suzanne G. Orchard, Alice J. Owen, Michelle A. Fravel, Julia Gilmartin‐Thomas, Joanne Ryan, Peter D. Fransquet, Rory Wolfe, Chenglong Yu, John J. McNeil, Paul Lacaze, Michael E. Ernst

**Affiliations:** ^1^ Department of Medical Genetics, Physiology & Pharmacology, and Psychiatry University of Calgary Calgary Alberta Canada; ^2^ The Mathison Centre for Mental Health Research & Education, Hotchkiss Brain Institute, Cumming School of Medicine University of Calgary Calgary Alberta Canada; ^3^ Alberta Children's Hospital Research Institute University of Calgary Calgary Alberta Canada; ^4^ Australian BioCommons The University of Melbourne Melbourne Victoria Australia; ^5^ School of Public Health and Preventive Medicine Monash University Melbourne Victoria Australia; ^6^ Department of Pharmacy Practice and Science, College of Pharmacy The University of Iowa Iowa City Iowa USA; ^7^ School of Allied Health, Human Services & Sport La Trobe University Melbourne Victoria Australia; ^8^ Allied Health Alfred Health Melbourne Victoria Australia; ^9^ Department of Family and Community Medicine, Carver College of Medicine The University of Iowa Iowa City Iowa USA

## Abstract

Older adults (65 years and over) frequently manage complex medication regimens and are vulnerable to adverse drug reactions and treatment inefficacies, some of which could be preventable with pharmacogenetics (PGx)‐guided prescribing. This study examined the prevalence of actionable PGx genotypes (i.e., those linked to a guideline that recommends a change to standard prescribing), the use of cautionary medications (i.e., those associated with an actionable PGx genotype), polypharmacy (i.e., ≥ 5 medications simultaneously), and cytochrome P450 enzyme inhibitor and inducer use among 13,670 older adults enrolled in the ASPirin in Reducing Events in the Elderly (ASPREE) trial. Genotyping was conducted for 10 pharmacogenes with actionable PGx‐based prescribing guidelines. Medication data were collected annually and assessed to identify cautionary medication use in the cohort. Most participants (98.8%) carried at least one actionable PGx genotype, with an average of three actionable genotypes per participant. *VKORC1* (61.1%) and *CYP2C19* (59.6%) were the most frequently observed genes with actionable genotypes. Statins (29.3%), nonsteroidal anti‐inflammatory drugs (14.2%), and proton‐pump inhibitors (7.9%) were the most used cautionary medications, with 27.5% of participants taking at least one medication for which PGx guidelines recommended a deviation from standard prescribing. Most (83.9%) participants reported taking a polypharmacy regimen, and 68.2% reported use of at least one cytochrome P450 enzyme inhibitor or inducer during the trial. Our findings underscore the high prevalence of actionable PGx genotypes, polypharmacy, and use of inhibitors and inducers in older adults, which collectively have the potential to inform safer and more effective prescribing practices.

**Study Highlights**

**WHAT IS THE CURRENT KNOWLEDGE ON THE TOPIC?**

Pharmacogenetic (PGx) testing can aid in personalized prescribing and may be particularly useful for older adults on complex medication regimens. Yet, the prevalence and clinical relevance of actionable PGx genotypes in older adults are not well characterized.

**WHAT QUESTION DID THIS STUDY ADDRESS?**

How common are actionable PGx genotypes (i.e., those linked to a guideline that recommends a change to standard prescribing), the use of cautionary medications (i.e., those associated with an actionable PGx genotype), polypharmacy (i.e., ≥ 5 medications simultaneously), and use of inhibitors and inducers of cytochrome 450 enzymes associated with guidelines in a relatively healthy, community‐dwelling older adult population? Additionally, what is the clinical actionability of PGx‐guided prescribing in this population?

**WHAT DOES THIS STUDY ADD TO OUR KNOWLEDGE?**

Nearly, all (98.8%) older adults carried at least one actionable PGx genotype, with 25.9% using at least one medication during the 5‐year study period that may benefit from PGx‐guided adjustments. Most reported polypharmacy (83.9%) and about two‐thirds (64.4%) reported the use of an inhibitor or inducer of a cytochrome P450 enzyme linked to a PGx‐based prescribing guideline.

**HOW MIGHT THIS CHANGE CLINICAL PHARMACOLOGY OR TRANSLATIONAL SCIENCE?**

These findings highlight the potential for PGx‐guided care in older adults, supporting safer, more effective prescribing for cautionary medications like statins, nonsteroidal anti‐inflammatory drugs, and proton‐pump inhibitors.

The foundation of current clinical pharmacogenetics (PGx) implementation lies in identifying genotypes associated with pharmacokinetic phenotypes (e.g., drug metabolism enzyme activity), which can predict drug efficacy and toxicity. These “actionable genotypes” have accompanying PGx‐based prescribing guidelines developed by expert groups to assist clinicians in the selection and dosing of over 160 medications.^1^, ^2^, ^3^

Identifying actionable PGx genotypes is particularly relevant for older adults (i.e., those aged 65 and older), who are often exposed to complex medication regimens and a high prevalence of polypharmacy (use of ≥ 5 medications simultaneously).^4^ This population is also highly vulnerable to adverse drug reactions and treatment inefficacies, many of which are preventable.^5^ PGx‐guided care represents a promising strategy to mitigate these risks in older adults. However, the extent to which older adults use medications associated with actionable PGx genotypes (i.e., cautionary medications) and the proportion whose PGx profiles are misaligned with their medication regimens remain poorly characterized.

In this study, we assessed a large cohort of community‐dwelling older adults and calculated the frequency of actionable PGx genotypes across 10 commonly tested pharmacogenes. We also provide estimates of the number of individuals taking cautionary medications and the proportion for whom deviations from standard prescribing practices would be advisable. Finally, we estimated the prevalence of polypharmacy and cytochrome P450 inhibitor and inducer use.

## METHODS

### Participants

This study included community‐dwelling adults in Australia and the United States (U.S.) aged 70 years and over (65 years and over for U.S. minority groups) who were enrolled in the ASPirin in Reducing Events in the Elderly (ASPREE) randomized, double‐blinded, placebo‐controlled trial (NCT01038583) conducted from 2010 to 2017, with a median follow‐up of 4.7 years.^6^ The primary aim of the ASPREE trial was to determine whether daily low‐dose aspirin could prolong disability‐free survival, a composite of death, dementia, or persistent physical disability, and the details of these results have been published.^7^

### Medication data

Participants were seen annually for study follow‐up visits in addition to 3‐to‐6‐month telephone calls, conducted by trained study staff according to standard operating procedures. At baseline and each annual visit, participants’ prescription medications were collected and reviewed by ASPREE study staff and coded using the Anatomical Therapeutic Chemical (ATC) coding system as previously described.^8^ For this current analysis, all medication data were assessed for in‐trial use of 69 drugs that have prescribing guidelines developed by the Clinical Pharmacogenetics Implementation Consortium (CPIC) or Dutch Pharmacogenetics Working Group (DPWG) (**Table** **S1**).

### Genotyping, imputation, and genotype‐to‐phenotype translation

All participants were invited to participate in the ASPREE Healthy Aging Biobank substudy (in Australia) or the ASPREE Cancer Endpoints Study (in US), which included collection of biospecimens for medical and genetic research.^9^ Genotyping was performed using the Axiom 2.0 Precision Medicine Diversity Array (Thermo Fisher Scientific, Waltham, MA). Variant calling used a custom pipeline and imputation was performed using the TOPMed Imputation Server with TOPMed‐r2 reference panel.^10^ Prior to imputation, variants with a call rate < 95% or Hardy–Weinberg *P* < 0.000001 were excluded. Samples with high missingness (> 5%), relatedness (kinship coefficients ≥ 0.1), or discordant sex information were excluded. Post‐imputation, variants with an imputation quality score (*R* ^2^) < 0.3 were excluded from downstream analyses. Each participant's genetic ancestry was assigned by principal component analysis of genotype data, with reference populations from the 1000 genomes Project phase 3 serving as a benchmark for ancestry classification.

All variants within the boundaries of 10 pharmacogenes (*CYP2B6, CYP2C9, CYP2C19, CYP2D6, CYP3A5, DPYD, NUDT15, SLCO1B1, TPMT, VKORC1*), including 50 kb up‐ and down‐stream of the boundaries, were extracted from the imputed variant call format (VCF) file using BCFtools (v1.17). For *CYP2B6*, the unimputed VCF file was used because the imputation process removed multiallelic variants that are critical to haplotype calling for this gene. Phasing was performed on *CYP2B6* using Beagle (5.2_21Apr21.304).^11^ SNVs for each gene and participant were then mapped to haplotype (star allele) definition tables obtained via the Pharmacogene Variation (PharmVar) Consortium's API.^12^ *TPMT and VKORC1* are not included in PharmVar, as such allele definition tables from the Pharmacogenomics Knowledge Base (PharmGKB) were utilized.^13^ Genotype‐to‐phenotype translation was performed according to diplotype‐phenotype tables available for each of the 10 examined genes on the PharmGKB website (**Supplementary Methods**).^13^ For *CYP2D6*, copy number variants could not be reliably detected and as such gene deletion (*5), duplication (*xN), and hybrid (e.g., *13, *36, *68) variants were not included in the analysis.

### Analysis

For each gene, we calculated the proportion of participants with an actionable genotype, defined as a genotype associated with one or more CPIC or DPWG prescribing recommendations that deviated from standard prescribing (i.e., a recommended dose adjustment or drug avoidance action). In addition, we calculated the number of participants who were using a cautionary medication at any study during the trial period, defined as a medication with an actionable CPIC or DPWG guideline. Among those reporting use of a cautionary medication(s), we estimated the proportion that a dose adjustment or alternative medication would be recommended, according to the CPIC and DPWG guidelines for the participant's genotype. Actionable genotype‐predicted phenotypes for each cautionary medication are listed in **Table** **S1**. Finally, we calculated the prevalence of polypharmacy (use of ≥ 5 drugs simultaneously)^4^ and the number of CYP2B6, CYP2C9, CYP2C19, CYP2D6, and CYP3A5 inhibitors and inducers reported by participants throughout the course of the trial. Inhibitors and inducers were defined according to the Drug Interactions Flockhart Table (https://drug‐interactions.medicine.iu.edu/MainTable.aspx).

### Ethics statement

The study was approved by local institutional review boards at each site and was conducted according to the principles of the Declaration of Helsinki. Inclusion and exclusion criteria have been described in detail elsewhere.^6^ Briefly, all participants were free from documented evidence of major cardiovascular disease, dementia, and significant independence‐limiting physical disability at enrollment and free of any concurrent illness expected to limit life expectancy to less than 5 years. Participants also could not have any conditions that posed a high risk of bleeding nor be currently using anticoagulants. All participants provided written informed consent prior to enrollment.

## RESULTS

Among the 19,114 participants enrolled in the ASPREE cohort, 13,776 (12,677 Australia/1,099 US) samples were genotyped, and 13,670 passed quality control. Medication data and genotype data were available for 12,912 participants. As such, the actionable genotype frequency analyses had a total sample of 13,670, while medication analyses had a total sample of 12,912. Most participants (95.9%, *n* = 13,119) were assigned to the European genetic ancestry group.

### Actionable genotype frequencies

**Figure 1:**
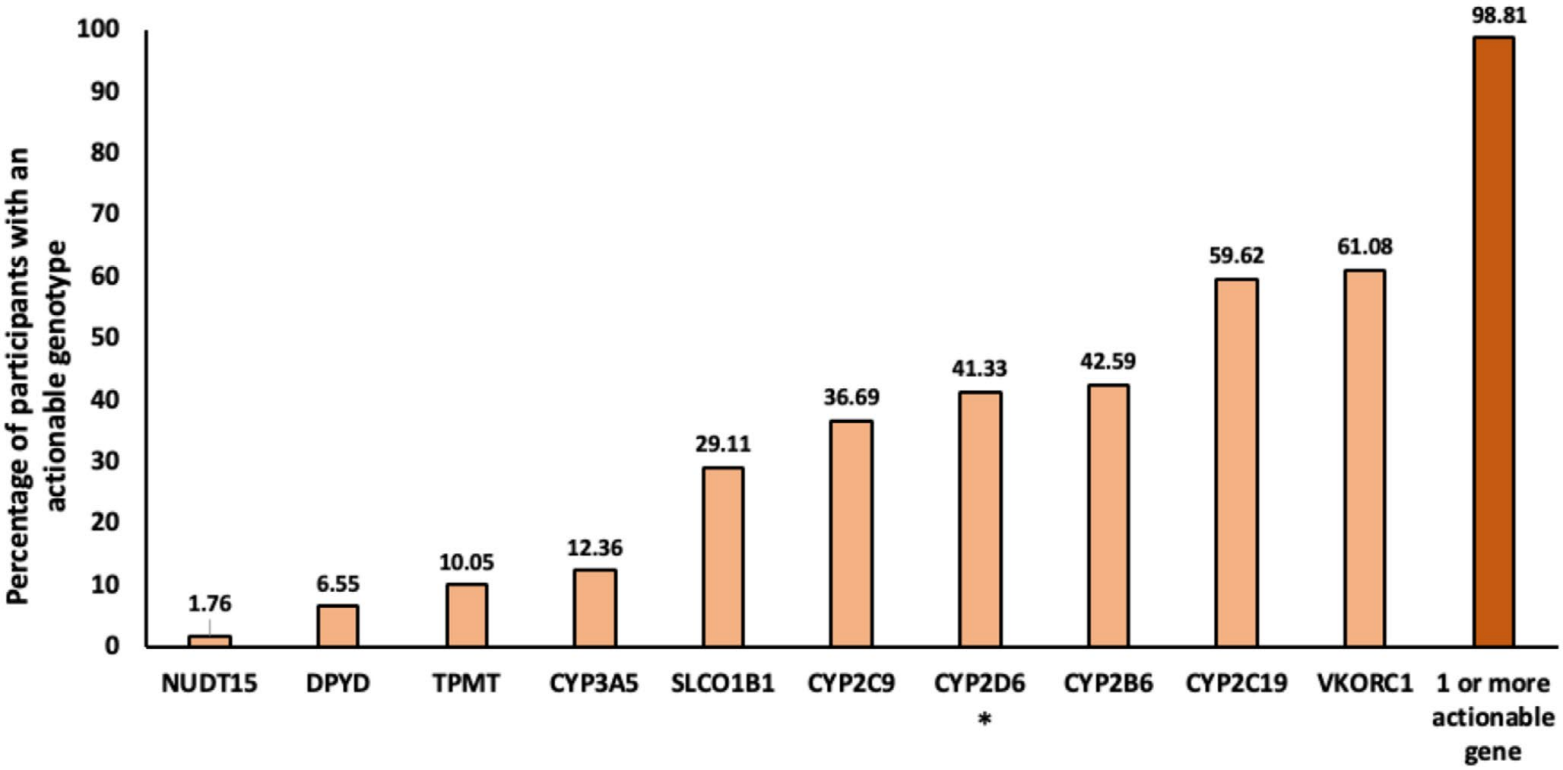
Percentage of participants with an actionable genotype by gene. *CYP2D6 copy number and structural variants (e.g., *xN, *5, *13, *36, *68) were not detectable, which could impact the percentage of participants with an actionable genotype.

Nearly all participants (98.8%, *n* = 13,507) had an actionable genotype for one or more of the 10 examined genes (**Figure** **1**). On average, participants carried 3.04 (standard deviation = 1.20) actionable genotypes (**Figure** **S1**). The highest proportion of participants had *VKORC1* (61.1%) and *CYP2C19* (59.6%) and the lowest had *NUDT15* (1.8%) and *DPYD* (6.5%) actionable genotypes. Detected genotypes, haplotypes, and predicted phenotype frequencies for each gene examined are provided in **Tables** **S2** **–** **S4**.

### Cautionary mediation use

**Figure 2:**
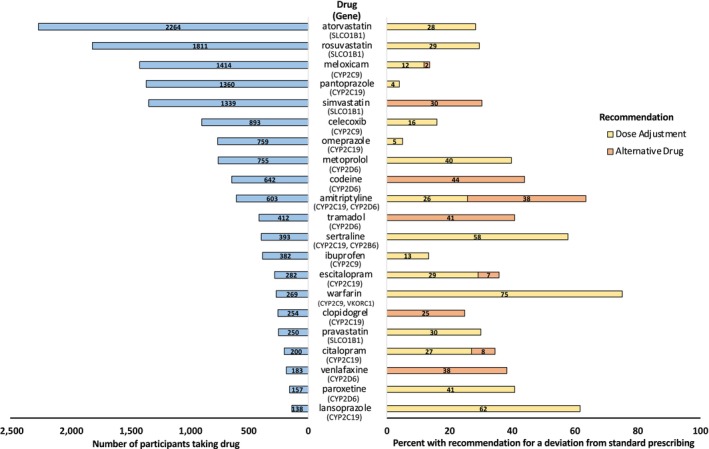
Cautionary medications used by 1% or more of ASPREE participants (left) and among those participants, the proportion with an actionable PGx genotype that current guidelines would recommend a dose adjustment or alternative drug (right). The proportion of participants with a CYP2D6 actionable genotype could be imprecise because copy number and structural variants (e.g., *xN, *5, *13, *36, *68) were not detectable.

Of the 69 cautionary medications examined, use of 49 (71.0%) was reported by one or more participants (**Table** **S5**) throughout the course of the trial. In total, 69.5% (*n* = 8,969) of participants reported use of a cautionary medication and 27.5% (*n* = 3,554) reported taking a cautionary medication for which a deviation from standard prescribing would be recommended, based on their PGx profile. Statins (i.e., atorvastatin, rosuvastatin, simvastatin) comprised three of the top five cautionary medications reported by participants and among those taking them, between 28 and 30% had actionable genotypes (**Figure** **2**). Use of other cautionary medications, such as warfarin, lansoprazole, amitriptyline, and sertraline, was reported less frequently but had the highest proportion (58–75%) of users that had actionable genotypes. Moreover, between 2% (meloxicam) and 44% (codeine) of participants reported use of a medication that current PGx guidelines would recommend avoidance based on their genotype (**Figure** **2**).

### Polypharmacy and use of inhibitors and inducers

Polypharmacy occurred during the trial in 83.9% (*n* = 10,844) participants. Among those on polypharmacy regimens, 70.0% (*n* = 7,591) reported use of at least one cautionary medication during the trial, whereas cautionary medication use among those without polypharmacy was 66.6% (*n* = 1,378). Moreover, 68.2% (*n* = 8,812) of participants reported taking at least one inhibitor or inducer of CYP2B6, CYP2C9, CYP2C19, CYP2D6, and CYP3A5 during the trial. Commonly used (> 1%) inhibitors were esomeprazole (28.7%, CYP2C19 moderate inhibitor), amlodipine (22.9%, CYP3A5 moderate inhibitor), omeprazole (8.6%, CYP2C19 weak inhibitor), diltiazem (2.9%, CYP3A5 moderate inhibitor), verapamil (2.7%, CYP3A5 moderate inhibitor), paroxetine (1.8%, CYP2D6 strong inhibitor), metronidazole (1.3%, CYP2C9 moderate inhibitor), duloxetine (1.2%, CYP2D6 moderate inhibitor), and fluoxetine (1.2%, CYP2D6 strong inhibitor) (**Table** **S6**). The most common inducers were prednisone (10.5%) betamethasone (7.1%), methylprednisolone (2.2%), prednisolone (1.5%), and dexamethasone (1.4%), all of which are CYP3A5 weak inducers (**Table** **S6**).

## DISCUSSION

We analyzed PGx data from 13,670 older adults and found nearly all (98.8%) of them carried at least one actionable PGx genotype. Our findings are consistent with numerous younger population‐based studies demonstrating the high prevalence of actionable PGx genotypes. For example, 100% of participants in the UK Biobank had at least one actionable PGx variant,^14^ and a study within the U.S. Veterans Health Administration estimated that 99% of veterans possessed at least one such PGx genotype.^15^ Similarly, research on the Swiss population showed that 97.3% of participants carried at least one actionable PGx genotype.^16^

Importantly, having an actionable PGx genotype does not necessarily mean a change in prescribing will be required or that it will be clinically applicable for a specific individual. For an actionable PGx genotype to be clinically relevant, the individual must either be considering the use of, or currently using, a medication for which the actionable genotype applies. We found that two‐thirds (65.6%) of participants reported use of at least one cautionary medication over the 5‐year trial period, but only one in every four (25.9%) used a cautionary medication for which a deviation from standard prescribing would be recommended based on their PGx profile. Our estimate of clinical relevance is comparable to the 23.7% reported in the UK Biobank and slightly less than the 31% of Swiss adults that were prescribed a medication that could benefit from a dose adjustment or alternative medication.^16^, ^17^

Our estimate of clinical relevance is likely conservative for two reasons. First, polypharmacy was highly prevalent, and a large proportion of participants reported using an inhibitor or inducer of one or more of the cytochrome P450 enzymes we examined. As such, it is likely that many of these individuals experienced phenoconversion – that is, a mismatch between a person's genotype‐predicted metabolism and observed metabolism.^18^ Future work within the cohort is planned to comprehensively investigate the impact of phenoconversion on actionability estimates and clinical outcomes. Second, 21 cautionary medications have actionable recommendations for CYP2D6 ultrarapid metabolizers (**Table** **S2**), who could not be detected with our genotyping approach. Likewise, *CYP2D6* gene deletion (*5) and hybrid (e.g., *13, *36, *68) variants could not be detected, which likely led to an underrepresentation of intermediate and poor metabolizers in our cohort. Among populations of European descent (95.9% of ASPREE participants), about 2% of the population are ultrarapid metabolizers, 49% are normal, 38% are intermediate, 7% are poor, and 4% are indeterminate.^13^ In contrast, the proportion of CYP2D6 normal, intermediate, poor, and indeterminate metabolizers in our cohort were respectively 56.7%, 35.7%, 5.8%, and 1.8% (**Table** **S4**) – suggesting approximately 6% of our cohort may have been assigned to an incorrect CYP2D6 metabolizer group.

The most frequently used cautionary medications in our study population were statins, nonsteroidal anti‐inflammatory drugs (NSAIDs), and proton‐pump inhibitors (PPIs). These three medication classes consistently rank among the top five prescribed to older adults in the US, Canada, and Australia.^19^, ^20^ Among statins, atorvastatin, rosuvastatin, and simvastatin were the most commonly used, aligning with national data from Australia over the past 6 years, which show that atorvastatin and rosuvastatin are the two most frequently prescribed medications annually.^20^ Nearly, one‐third (29.3%) of participants who used statins carried one or more actionable genetic variants for which a dose adjustment or alternative statin would be recommended to reduce the risk of musculoskeletal symptoms.^21^ Future research within this cohort will compare rates of statin discontinuation and switching between participants with and without actionable variants, contributing to the evidence base for the clinical utility of PGx‐guided statin therapy.

One in five (20.4%) participants reported using an NSAID, among whom 14.2% carried an actionable PGx genotype. NSAIDs are the most used class of analgesics for the treatment of both acute and chronic pain.^22^ They are primarily metabolized by *CYP2C9*, and reduced enzymatic function (e.g., intermediate or poor metabolizers) can lead to higher plasma concentrations and elevated risk of toxicities, including gastrointestinal, renal, and cardiovascular adverse events. To date, seven NSAIDs (celecoxib, flurbiprofen, ibuprofen, lornoxicam, meloxicam, piroxicam, tenoxicam) have *CYP2C9* genotype‐based guidelines.^23^ Despite the availability of these guidelines, the real‐world application and outcomes of *CYP2C9*‐guided NSAID prescribing, particularly in older adults, are limited. Future research in this area could provide critical insights into optimizing pain management while minimizing harm in this vulnerable population.

PPIs were used by 16.6% of study participants, and 7.9% of these users carried an actionable genotype. CYP2C19 is responsible for about 80% of the clearance of dexlansoprazole, omeprazole, lansoprazole, and pantoprazole.^24^ Consequently, individuals with the ultrarapid metabolizer phenotype are at an increased risk of receiving limited therapeutic benefit from standard doses of these drugs.^24^ In older adults, PPIs are frequently used, but this use comes with a higher risk of adverse effects, which include electrolyte imbalances (e.g., hypomagnesemia), *bone fractures, chronic kidney disease*, and ***Clostridium difficile*** *infections*.^25^ These risks are further exacerbated by age‐related physiological changes, polypharmacy, and the presence of comorbidities. Given these complexities, CYP2C19‐informed PPI prescribing strategies could help optimize safety and efficacy.

Limitations of this study should be noted. The exclusion of *CYP2D6* copy number and structural variants, due to constraints of the genotyping platform, may have led to an underestimation of actionable genotypes for this gene. More recent genome‐wide genotyping platforms developed by Thermofisher and Illumina have addressed this constraint by inclusion of *CYP2D6* copy number and structural variants, which should improve *CYP2D6* actionability estimates in the future. Additionally, the ASPREE cohort comprises individuals predominantly of European background, which limits generalizability to more diverse populations, emphasizing the need for further research in underrepresented groups. Lastly, medication dose information was not collected, prohibiting further characterization of cautionary medication use.

In conclusion, our findings emphasize the substantial proportion of older adults who may benefit from PGx‐guided prescribing to enhance medication safety and effectiveness. While previous studies have extensively examined PGx implementation in clinical settings, our work uniquely focuses on community‐dwelling older adults, a group particularly vulnerable to polypharmacy and adverse drug events. The high prevalence of actionable PGx genotypes, cautionary medication use, polypharmacy, and use of cytochrome P450 inhibitors and inducers, along with favorable cost‐effectiveness demonstrated by others,^26^ highlights the potential for integrating PGx testing into routine clinical practice for older adults.

## Funding

Initial support to establish the Healthy Aging Biobank was through a Preventative Health Flagship 2009 research grant from the Australian Government's CSIRO (Commonwealth Scientific and Industrial Research Organization), for the baseline biospecimen collections from the first 8000 ASPREE participants (www.csiro.org/). The subsequent > 4,000 baseline collections and all year 3 collections were funded by the National Cancer Institute (grant number 5U01AG029824–02) at the US National Institutes of Health (https://www.nih/gov/). Monash University provided funds to establish the ultracold sample storage biorepository at the Alfred Hospital/Monash campus. Genotyping of the ASPREE cohort was supported by a Bioplatforms Australia National Framework Initiative grant (2018–2020). PL is supported by an NHMRC Leadership fellowship (2026325). JJMcN is supported by an NHMRC leadership fellowship [IG 1173690]. The ASPREE project was supported by grants (U01AG029824 and U19AG062682) from the National Institute on Aging and the National Cancer Institute at the National Institutes of Health, by grants (334047 and 1127060) from the National Health and Medical Research Council of Australia, and by Monash University and the Victorian Cancer Agency. The funders had no role in study design, data collection and analysis, decision to publish, or preparation of the manuscript. There was no additional external funding received for this study.

## Conflicts of interest

C. Bousman is the founder of Sequence2Script Inc. All other authors declared no competing interests for this work.

## Author contributions

C.A.B. and A.N. wrote the manuscript; R.L.W., S.G.O., A.J.O., M.A.F., J.G.‐T., J.R., P.D.F., R.W., C.Y., J.J.M., P.L., and M.E.E. designed the research; R.L.W., S.G.O., A.J.O., M.A.F., J.G.‐T., J.R., P.D.F., R.W., C.Y., J.J.M., P.L., and M.E.E. performed the research; A.N., C.A.B., and Z.A.B. analyzed the data.

## Supporting information


Data S1
