# Supplementary material for: Prevalence of Actionable Pharmacogenetic Genotype Frequencies, Cautionary Medication Use, and Polypharmacy in Community‐Dwelling Older Adults
Source: Clin Pharmacol Ther. 2025 Apr 30;118(2):337–42. doi: 10.1002/cpt.3702 (PMC12272320; doi:10.1002/cpt.3702)
Supplement: Supplementary file 1 — Data S1 [file CPT-118-337-s001.docx]

**SUPPLEMENTARY METHODS**

Date sources used for allele definition and diplotype-to-phenotype translation (retrieved Jan 8, 2025).

*CYP2B6,* [*https://www.pharmgkb.org/page/cyp2b6RefMaterials*](https://www.pharmgkb.org/page/cyp2b6RefMaterials)

*CYP2C9,* [*https://www.pharmgkb.org/page/cyp2c9RefMaterials*](https://www.pharmgkb.org/page/cyp2c9RefMaterials)

*CYP2C19,* [*https://www.pharmgkb.org/page/cyp2c19RefMaterials*](https://www.pharmgkb.org/page/cyp2c19RefMaterials)

*CYP2D6,* [*https://www.pharmgkb.org/page/cyp2d6RefMaterials*](https://www.pharmgkb.org/page/cyp2d6RefMaterials)

*CYP3A5,* [*https://www.pharmgkb.org/page/cyp3a5RefMaterials*](https://www.pharmgkb.org/page/cyp3a5RefMaterials)

*DPYD,* [*https://www.pharmgkb.org/page/dpydRefMaterials*](https://www.pharmgkb.org/page/dpydRefMaterials)

*NUDT15,* [*https://www.pharmgkb.org/page/nudt15RefMaterials*](https://www.pharmgkb.org/page/nudt15RefMaterials)

*SLCO1B1,* [*https://www.pharmgkb.org/page/slco1b1RefMaterials*](https://www.pharmgkb.org/page/slco1b1RefMaterials)

*TPMT,* [*https://www.pharmgkb.org/page/tpmtRefMaterials*](https://www.pharmgkb.org/page/tpmtRefMaterials)

*VKORC1,* [*https://www.pharmgkb.org/page/vkorc1RefMaterials*](https://www.pharmgkb.org/page/vkorc1RefMaterials)

**Figure S1.** Distribution of total number of actionable genotypes per person in the ASPREE cohort. Mean = 3.04, median = 3.00, standard deviation = 1.20

**Supplementary Table S1.** Drugs, Genes, and Actionable Phenotypes

|  |  | Actionable Phenotypes |  |  |
| --- | --- | --- | --- | --- |
| Drug | Gene | CPIC | DPWG |  |
|  |  |  |  |  |
| Acenocoumarol | VKORC1 | – | Low (rs9923231 AA) |  |
| Amitriptyline | CYP2C19 | UM, RM, PM* | – |  |
| Amitriptyline ^ | CYP2D6 | UM, PM | UM, IM, PM |  |
| Aripiprazole | CYP2D6 | – | PM |  |
| Atomoxetine ^ | CYP2D6 | IM, PM | UM, IM, PM |  |
| Atorvastatin | SLCO1B1 | Poor*, Decreased* | Poor (rs4149056 CC), Decreased (rs4149056 TC) |  |
| Azathioprine | NUDT15 | IM*, PM | IM, PM |  |
| Azathioprine | TPMT | IM*, PM | IM, PM |  |
| Brexpiprazole | CYP2D6 | – | PM |  |
| Capecitabine | DPYD | IM, PM | IM, PM |  |
| Celecoxib | CYP2C9 | PM, IM (AS=1) | – |  |
| Citalopram | CYP2C19 | UM, IM, PM* | IM, PM |  |
| Clomipramine | CYP2C19 | UM, RM, IM*, PM* | UM |  |
| Clomipramine ^ | CYP2D6 | UM, IM, PM | UM, IM, PM |  |
| Clopidogrel | CYP2C19 | IM*, PM* | IM, PM |  |
| Codeine ^ | CYP2D6 | UM, IM, PM | UM, IM, PM |  |
| Desipramine ^ | CYP2D6 | UM, IM, PM | – |  |
| Dexlansoprazole | CYP2C19 | UM | – |  |
| Doxepin | CYP2C19 | UM, RM, IM*, PM* | – |  |
| Doxepin ^ | CYP2D6 | UM, IM, PM | UM, IM, PM |  |
| Efavirenz | CYP2B6 | IM, PM | IM, PM |  |
| Eliglustat ^ | CYP2D6 | – | UM, IM, PM |  |
| Escitalopram | CYP2C19 | UM, IM, PM* | UM, IM, PM |  |
| Flecainide ^ | CYP2D6 | – | UM, IM, PM |  |
| Flucytosine | DPYD | – | PM |  |
| Fluorouracil | DPYD | IM, PM | IM, PM |  |
| Flurbiprofen | CYP2C9 | PM, IM (AS=1) | – |  |
| Fluvastatin | SLCO1B1 | Poor*, Decreased* | – |  |
| Fluvastatin | CYP2C9 | PM, IM | – |  |
| Fluvoxamine | CYP2D6 | PM | – |  |
| Fosphenytoin | CYP2C9 | PM, IM (AS=1) | – |  |
| Haloperidol | CYP2D6 | – | PM |  |
| Hydrocodone | CYP2D6 | PM, IM | – |  |
| Ibuprofen | CYP2C9 | PM, IM (AS=1) | – |  |
| Imipramine | CYP2C19 | UM, RM, PM* | PM |  |
| Imipramine ^ | CYP2D6 | UM, IM, PM | UM, IM, PM |  |
| Lansoprazole | CYP2C19 | UM, RM, IM*, PM* | UM |  |
| Lornoxicam | CYP2C9 | PM | – |  |
| Lovastatin | SLCO1B1 | Poor*, Decreased* | – |  |
| Meloxicam | CYP2C9 | PM, IM (AS=1) | – |  |
| Mercaptopurine | NUDT15 | IM, PM | IM, PM |  |
| Mercaptopurine | TPMT | IM, PM | IM, PM |  |
| Metoprolol ^ | CYP2D6 | PM | UM, IM, PM |  |
| Nortriptyline ^ | CYP2D6 | UM, IM, PM | UM, IM, PM |  |
| Omeprazole | CYP2C19 | UM | UM |  |
| Ondansetron ^ | CYP2D6 | UM | – |  |
| Pantoprazole | CYP2C19 | UM | UM |  |
| Paroxetine ^ | CYP2D6 | UM, IM, PM | UM |  |
| Phenprocoumon | VKORC1 | – | Low (rs9923231 AA) |  |
| Phenytoin | CYP2C9 | PM, IM (AS=1) | PM, IM |  |
| Pimozide | CYP2D6 | – | IM, PM |  |
| Piroxicam | CYP2C9 | PM, IM (AS=1) | – |  |
| Pitavastatin | SLCO1B1 | Poor*, Decreased* | – |  |
| Pravastatin | SLCO1B1 | Poor*, Decreased* |  |  |
| Propafenone ^ | CYP2D6 | – | UM, IM, PM |  |
| Quetiapine | CYP3A4 | – | PM |  |
| Risperidone ^ | CYP2D6 | – | UM, PM |  |
| Rosuvastatin | SLCO1B1 | Poor*, Decreased* | Poor (rs4149056 CC), Decreased (rs4149056 TC) |  |
| Sertraline | CYP2C19 | IM*, PM* | PM |  |
| Sertraline | CYP2B6 | IM, PM | – |  |
| Simvastatin | SLCO1B1 | Poor*, Decreased* | Poor (rs4149056 CC), Decreased (rs4149056 TC) |  |
| Siponimod | CYP2C9 | – | IM, PM |  |
| Tacrolimus | CYP3A5 | IM, NM | IM, NM |  |
| Tamoxifen | CYP2D6 | IM, PM | IM, PM |  |
| Tegafur | DPYD | – | PM, IM |  |
| Tenoxicam | CYP2C9 | PM, IM (AS=1) | – |  |
| Thioguanine | TPMT | IM, PM | IM, PM |  |
| Thioguanine | NUDT15 | IM, PM | IM, PM |  |
| Tramadol ^ | CYP2D6 | UM, IM, PM | UM, IM, PM |  |
| Trimipramine | CYP2C19 | UM, RM, PM* | – |  |
| Trimipramine ^ | CYP2D6 | UM, IM, PM | – |  |
| Tropisetron ^ | CYP2D6 | UM | – |  |
| Venlafaxine ^ | CYP2D6 | PM | IM, PM |  |
| Voriconazole | CYP2C19 | UM, RM, PM | UM, IM, PM |  |
| Vortioxetine ^ | CYP2D6 | UM, PM | – |  |
| Warfarin | CYP2C9 | PM (*2/*2, *3/*3), IM (*2/*3) | PM, IM |  |
| Warfarin | VKORC1 | Low (rs9923231 AA)*, Intermediate (rs9923231 AG)* | Low (rs9923231 AA) |  |
| Zuclopenthixol ^ | CYP2D6 | – | UM, IM, PM |  |
| ^ Drugs with an actionable recommendation associated with CYP2D6 UM genotypes, for which we could not detect in this study due to limitations of the genotyping platform. * Includes likely or possible phenotypes defined in CPIC genotype-to-phenotype translation tables; Underlined text = alternative drug recommended, else dose adjustment is indicated. AS, activity score; CPIC, Clinical Pharmacogenetics Implementation Consortium; DPWG, Dutch Pharmacogenetics Working Group; IM, intermediate metabolizer; NM, normal metabolizer; PM, poor metabolizer; RM, rapid metabolizer; UM, ultrarapid metabolizer | | | |  |

| **Supplementary Table S2.** Detected genotype or diplotype frequencies | | | | | | | | | | | | | | | | |  |
| --- | --- | --- | --- | --- | --- | --- | --- | --- | --- | --- | --- | --- | --- | --- | --- | --- | --- |
|  | Genotype or Diplotype | Phenotype | Full Sample  (13,670) | | EUR (13,119) | | SAS (60) | | AMR (56) | | AFR (8) | | Mixed (46) | | Unknown  (381) | |  |
| Gene |  |  | N | % | N | % | N | % | N | % | N | % | N | % | N | % |  |
| CYP2B6 | *1\|*1 | NM | 4121 | 30.1 | 3966 | 30.2 | 10 | 16.7 | 20 | 35.7 | 3 | 37.5 | 13 | 28.3 | 109 | 28.6 |  |
| CYP2B6 | *11\|*13 | IND | 1 | 0 | 1 | 0 | 0 | 0 | 0 | 0 | 0 | 0 | 0 | 0 | 0 | 0 |  |
| CYP2B6 | *11\|*15 | IND | 2 | 0 | 2 | 0 | 0 | 0 | 0 | 0 | 0 | 0 | 0 | 0 | 0 | 0 |  |
| CYP2B6 | *11\|*2 | IND | 5 | 0 | 4 | 0 | 0 | 0 | 0 | 0 | 0 | 0 | 0 | 0 | 1 | 0.3 |  |
| CYP2B6 | *11\|*5 | IND | 18 | 0.1 | 17 | 0.1 | 0 | 0 | 0 | 0 | 0 | 0 | 0 | 0 | 1 | 0.3 |  |
| CYP2B6 | *11\|*6 | IND | 30 | 0.2 | 29 | 0.2 | 0 | 0 | 0 | 0 | 0 | 0 | 0 | 0 | 1 | 0.3 |  |
| CYP2B6 | *12\|*2 | IM | 1 | 0 | 1 | 0 | 0 | 0 | 0 | 0 | 0 | 0 | 0 | 0 | 0 | 0 |  |
| CYP2B6 | *12\|*5 | IM | 1 | 0 | 1 | 0 | 0 | 0 | 0 | 0 | 0 | 0 | 0 | 0 | 0 | 0 |  |
| CYP2B6 | *12\|*6 | PM | 1 | 0 | 1 | 0 | 0 | 0 | 0 | 0 | 0 | 0 | 0 | 0 | 0 | 0 |  |
| CYP2B6 | *13\|*15 | IND | 2 | 0 | 2 | 0 | 0 | 0 | 0 | 0 | 0 | 0 | 0 | 0 | 0 | 0 |  |
| CYP2B6 | *13\|*2 | IM | 6 | 0 | 5 | 0 | 0 | 0 | 0 | 0 | 0 | 0 | 0 | 0 | 1 | 0.3 |  |
| CYP2B6 | *13\|*22 | IND | 2 | 0 | 2 | 0 | 0 | 0 | 0 | 0 | 0 | 0 | 0 | 0 | 0 | 0 |  |
| CYP2B6 | *13\|*4 | IM | 3 | 0 | 3 | 0 | 0 | 0 | 0 | 0 | 0 | 0 | 0 | 0 | 0 | 0 |  |
| CYP2B6 | *13\|*5 | IM | 18 | 0.1 | 17 | 0.1 | 1 | 1.7 | 0 | 0 | 0 | 0 | 0 | 0 | 0 | 0 |  |
| CYP2B6 | *13\|*6 | PM | 27 | 0.2 | 26 | 0.2 | 0 | 0 | 0 | 0 | 0 | 0 | 0 | 0 | 1 | 0.3 |  |
| CYP2B6 | *15\|*15 | IND | 1 | 0 | 1 | 0 | 0 | 0 | 0 | 0 | 0 | 0 | 0 | 0 | 0 | 0 |  |
| CYP2B6 | *15\|*2 | IND | 13 | 0.1 | 13 | 0.1 | 0 | 0 | 0 | 0 | 0 | 0 | 0 | 0 | 0 | 0 |  |
| CYP2B6 | *15\|*22 | IND | 5 | 0 | 4 | 0 | 0 | 0 | 0 | 0 | 0 | 0 | 0 | 0 | 1 | 0.3 |  |
| CYP2B6 | *15\|*4 | IND | 5 | 0 | 5 | 0 | 0 | 0 | 0 | 0 | 0 | 0 | 0 | 0 | 0 | 0 |  |
| CYP2B6 | *15\|*5 | IND | 36 | 0.3 | 34 | 0.3 | 0 | 0 | 0 | 0 | 0 | 0 | 0 | 0 | 2 | 0.5 |  |
| CYP2B6 | *15\|*6 | IND | 60 | 0.4 | 57 | 0.4 | 0 | 0 | 0 | 0 | 0 | 0 | 0 | 0 | 3 | 0.8 |  |
| CYP2B6 | *18\|*6 | PM | 2 | 0 | 0 | 0 | 0 | 0 | 0 | 0 | 2 | 25 | 0 | 0 | 0 | 0 |  |
| CYP2B6 | *1\|*11 | IND | 53 | 0.4 | 53 | 0.4 | 0 | 0 | 0 | 0 | 0 | 0 | 0 | 0 | 0 | 0 |  |
| CYP2B6 | *1\|*12 | IM | 1 | 0 | 1 | 0 | 0 | 0 | 0 | 0 | 0 | 0 | 0 | 0 | 0 | 0 |  |
| CYP2B6 | *1\|*13 | IM | 73 | 0.5 | 72 | 0.5 | 0 | 0 | 0 | 0 | 0 | 0 | 0 | 0 | 1 | 0.3 |  |
| CYP2B6 | *1\|*15 | IND | 138 | 1 | 133 | 1 | 0 | 0 | 0 | 0 | 0 | 0 | 0 | 0 | 5 | 1.3 |  |
| CYP2B6 | *1\|*2 | NM | 678 | 5 | 647 | 4.9 | 3 | 5 | 2 | 3.6 | 1 | 12.5 | 4 | 8.7 | 21 | 5.5 |  |
| CYP2B6 | *1\|*22 | IND | 125 | 0.9 | 124 | 0.9 | 0 | 0 | 0 | 0 | 0 | 0 | 0 | 0 | 1 | 0.3 |  |
| CYP2B6 | *1\|*26 | IM | 1 | 0 | 1 | 0 | 0 | 0 | 0 | 0 | 0 | 0 | 0 | 0 | 0 | 0 |  |
| CYP2B6 | *1\|*36 | IM | 8 | 0.1 | 8 | 0.1 | 0 | 0 | 0 | 0 | 0 | 0 | 0 | 0 | 0 | 0 |  |
| CYP2B6 | *1\|*38 | IM | 1 | 0 | 1 | 0 | 0 | 0 | 0 | 0 | 0 | 0 | 0 | 0 | 0 | 0 |  |
| CYP2B6 | *1\|*4 | RM | 241 | 1.8 | 232 | 1.8 | 1 | 1.7 | 0 | 0 | 0 | 0 | 1 | 2.2 | 7 | 1.8 |  |
| CYP2B6 | *1\|*5 | NM | 1803 | 13.2 | 1744 | 13.3 | 7 | 11.7 | 5 | 8.9 | 0 | 0 | 3 | 6.5 | 44 | 11.5 |  |
| CYP2B6 | *1\|*6 | IM | 3581 | 26.2 | 3413 | 26 | 24 | 40 | 20 | 35.7 | 2 | 25 | 18 | 39.1 | 104 | 27.3 |  |
| CYP2B6 | *1\|*7 | IM | 6 | 0 | 6 | 0 | 0 | 0 | 0 | 0 | 0 | 0 | 0 | 0 | 0 | 0 |  |
| CYP2B6 | *1\|*8 | IM | 3 | 0 | 3 | 0 | 0 | 0 | 0 | 0 | 0 | 0 | 0 | 0 | 0 | 0 |  |
| CYP2B6 | *1\|*9 | IM | 53 | 0.4 | 50 | 0.4 | 0 | 0 | 0 | 0 | 0 | 0 | 0 | 0 | 3 | 0.8 |  |
| CYP2B6 | *22\|*4 | IND | 3 | 0 | 3 | 0 | 0 | 0 | 0 | 0 | 0 | 0 | 0 | 0 | 0 | 0 |  |
| CYP2B6 | *22\|*5 | IND | 37 | 0.3 | 35 | 0.3 | 0 | 0 | 0 | 0 | 0 | 0 | 0 | 0 | 2 | 0.5 |  |
| CYP2B6 | *22\|*6 | IND | 66 | 0.5 | 64 | 0.5 | 0 | 0 | 0 | 0 | 0 | 0 | 0 | 0 | 2 | 0.5 |  |
| CYP2B6 | *22\|*9 | IND | 1 | 0 | 1 | 0 | 0 | 0 | 0 | 0 | 0 | 0 | 0 | 0 | 0 | 0 |  |
| CYP2B6 | *2\|*2 | NM | 32 | 0.2 | 31 | 0.2 | 0 | 0 | 0 | 0 | 0 | 0 | 1 | 2.2 | 0 | 0 |  |
| CYP2B6 | *2\|*22 | IND | 9 | 0.1 | 9 | 0.1 | 0 | 0 | 0 | 0 | 0 | 0 | 0 | 0 | 0 | 0 |  |
| CYP2B6 | *2\|*4 | RM | 21 | 0.2 | 21 | 0.2 | 0 | 0 | 0 | 0 | 0 | 0 | 0 | 0 | 0 | 0 |  |
| CYP2B6 | *2\|*5 | NM | 142 | 1 | 133 | 1 | 0 | 0 | 1 | 1.8 | 0 | 0 | 1 | 2.2 | 7 | 1.8 |  |
| CYP2B6 | *2\|*6 | IM | 275 | 2 | 266 | 2 | 1 | 1.7 | 2 | 3.6 | 0 | 0 | 0 | 0 | 6 | 1.6 |  |
| CYP2B6 | *2\|*9 | IM | 2 | 0 | 2 | 0 | 0 | 0 | 0 | 0 | 0 | 0 | 0 | 0 | 0 | 0 |  |
| CYP2B6 | *36\|*4 | IM | 1 | 0 | 1 | 0 | 0 | 0 | 0 | 0 | 0 | 0 | 0 | 0 | 0 | 0 |  |
| CYP2B6 | *36\|*5 | IM | 2 | 0 | 2 | 0 | 0 | 0 | 0 | 0 | 0 | 0 | 0 | 0 | 0 | 0 |  |
| CYP2B6 | *36\|*6 | PM | 1 | 0 | 1 | 0 | 0 | 0 | 0 | 0 | 0 | 0 | 0 | 0 | 0 | 0 |  |
| CYP2B6 | *4\|*4 | UM | 5 | 0 | 4 | 0 | 0 | 0 | 0 | 0 | 0 | 0 | 1 | 2.2 | 0 | 0 |  |
| CYP2B6 | *4\|*5 | RM | 55 | 0.4 | 54 | 0.4 | 1 | 1.7 | 0 | 0 | 0 | 0 | 0 | 0 | 0 | 0 |  |
| CYP2B6 | *4\|*6 | IM | 119 | 0.9 | 116 | 0.9 | 0 | 0 | 1 | 1.8 | 0 | 0 | 0 | 0 | 2 | 0.5 |  |
| CYP2B6 | *4\|*9 | IM | 2 | 0 | 2 | 0 | 0 | 0 | 0 | 0 | 0 | 0 | 0 | 0 | 0 | 0 |  |
| CYP2B6 | *5\|*5 | NM | 207 | 1.5 | 197 | 1.5 | 0 | 0 | 0 | 0 | 0 | 0 | 1 | 2.2 | 9 | 2.4 |  |
| CYP2B6 | *5\|*6 | IM | 752 | 5.5 | 723 | 5.5 | 2 | 3.3 | 2 | 3.6 | 0 | 0 | 1 | 2.2 | 24 | 6.3 |  |
| CYP2B6 | *5\|*9 | IM | 9 | 0.1 | 9 | 0.1 | 0 | 0 | 0 | 0 | 0 | 0 | 0 | 0 | 0 | 0 |  |
| CYP2B6 | *6\|*6 | PM | 798 | 5.8 | 760 | 5.8 | 10 | 16.7 | 3 | 5.4 | 0 | 0 | 2 | 4.3 | 23 | 6 |  |
| CYP2B6 | *6\|*7 | PM | 4 | 0 | 4 | 0 | 0 | 0 | 0 | 0 | 0 | 0 | 0 | 0 | 0 | 0 |  |
| CYP2B6 | *6\|*8 | PM | 1 | 0 | 1 | 0 | 0 | 0 | 0 | 0 | 0 | 0 | 0 | 0 | 0 | 0 |  |
| CYP2B6 | *9\|*9 | PM | 1 | 0 | 1 | 0 | 0 | 0 | 0 | 0 | 0 | 0 | 0 | 0 | 0 | 0 |  |
| CYP2C19 | *1\|*1 | NM | 5083 | 37.2 | 4897 | 37.3 | 7 | 11.7 | 26 | 46.4 | 3 | 37.5 | 12 | 26.1 | 138 | 36.2 |  |
| CYP2C19 | *10\|*2 | PM | 1 | 0 | 1 | 0 | 0 | 0 | 0 | 0 | 0 | 0 | 0 | 0 | 0 | 0 |  |
| CYP2C19 | *13\|*15 | NM | 2 | 0 | 2 | 0 | 0 | 0 | 0 | 0 | 0 | 0 | 0 | 0 | 0 | 0 |  |
| CYP2C19 | *15\|*15 | NM | 10 | 0.1 | 10 | 0.1 | 0 | 0 | 0 | 0 | 0 | 0 | 0 | 0 | 0 | 0 |  |
| CYP2C19 | *15\|*17 | RM | 146 | 1.1 | 141 | 1.1 | 1 | 1.7 | 1 | 1.8 | 0 | 0 | 1 | 2.2 | 2 | 0.5 |  |
| CYP2C19 | *15\|*2 | IM | 9 | 0.1 | 6 | 0 | 1 | 1.7 | 0 | 0 | 0 | 0 | 1 | 2.2 | 1 | 0.3 |  |
| CYP2C19 | *15\|*8 | IM | 18 | 0.1 | 17 | 0.1 | 0 | 0 | 0 | 0 | 0 | 0 | 0 | 0 | 1 | 0.3 |  |
| CYP2C19 | *17\|*17 | UM | 656 | 4.8 | 641 | 4.9 | 0 | 0 | 2 | 3.6 | 0 | 0 | 1 | 2.2 | 12 | 3.1 |  |
| CYP2C19 | *17\|*2 | IM | 850 | 6.2 | 815 | 6.2 | 5 | 8.3 | 0 | 0 | 1 | 12.5 | 5 | 10.9 | 24 | 6.3 |  |
| CYP2C19 | *17\|*3 | IM | 1 | 0 | 0 | 0 | 1 | 1.7 | 0 | 0 | 0 | 0 | 0 | 0 | 0 | 0 |  |
| CYP2C19 | *17\|*35 | IM | 3 | 0 | 2 | 0 | 0 | 0 | 0 | 0 | 0 | 0 | 0 | 0 | 1 | 0.3 |  |
| CYP2C19 | *17\|*39 | IND | 1 | 0 | 1 | 0 | 0 | 0 | 0 | 0 | 0 | 0 | 0 | 0 | 0 | 0 |  |
| CYP2C19 | *17\|*4 | IM | 11 | 0.1 | 11 | 0.1 | 0 | 0 | 0 | 0 | 0 | 0 | 0 | 0 | 0 | 0 |  |
| CYP2C19 | *17\|*8 | IM | 15 | 0.1 | 15 | 0.1 | 0 | 0 | 0 | 0 | 0 | 0 | 0 | 0 | 0 | 0 |  |
| CYP2C19 | *17\|*9 | IM | 1 | 0 | 1 | 0 | 0 | 0 | 0 | 0 | 0 | 0 | 0 | 0 | 0 | 0 |  |
| CYP2C19 | *1\|*15 | NM | 419 | 3.1 | 399 | 3 | 3 | 5 | 2 | 3.6 | 0 | 0 | 2 | 4.3 | 13 | 3.4 |  |
| CYP2C19 | *1\|*17 | RM | 3476 | 25.4 | 3349 | 25.5 | 10 | 16.7 | 15 | 26.8 | 1 | 12.5 | 7 | 15.2 | 94 | 24.7 |  |
| CYP2C19 | *1\|*18 | NM | 1 | 0 | 1 | 0 | 0 | 0 | 0 | 0 | 0 | 0 | 0 | 0 | 0 | 0 |  |
| CYP2C19 | *1\|*2 | IM | 2586 | 18.9 | 2456 | 18.7 | 20 | 33.3 | 9 | 16.1 | 3 | 37.5 | 16 | 34.8 | 82 | 21.5 |  |
| CYP2C19 | *1\|*22 | IM | 1 | 0 | 1 | 0 | 0 | 0 | 0 | 0 | 0 | 0 | 0 | 0 | 0 | 0 |  |
| CYP2C19 | *1\|*3 | IM | 2 | 0 | 2 | 0 | 0 | 0 | 0 | 0 | 0 | 0 | 0 | 0 | 0 | 0 |  |
| CYP2C19 | *1\|*30 | IND | 2 | 0 | 2 | 0 | 0 | 0 | 0 | 0 | 0 | 0 | 0 | 0 | 0 | 0 |  |
| CYP2C19 | *1\|*35 | IM | 2 | 0 | 2 | 0 | 0 | 0 | 0 | 0 | 0 | 0 | 0 | 0 | 0 | 0 |  |
| CYP2C19 | *1\|*39 | IND | 1 | 0 | 1 | 0 | 0 | 0 | 0 | 0 | 0 | 0 | 0 | 0 | 0 | 0 |  |
| CYP2C19 | *1\|*4 | IM | 20 | 0.1 | 20 | 0.2 | 0 | 0 | 0 | 0 | 0 | 0 | 0 | 0 | 0 | 0 |  |
| CYP2C19 | *1\|*6 | IM | 2 | 0 | 2 | 0 | 0 | 0 | 0 | 0 | 0 | 0 | 0 | 0 | 0 | 0 |  |
| CYP2C19 | *1\|*8 | IM | 23 | 0.2 | 23 | 0.2 | 0 | 0 | 0 | 0 | 0 | 0 | 0 | 0 | 0 | 0 |  |
| CYP2C19 | *2\|*2 | PM | 295 | 2.2 | 271 | 2.1 | 12 | 20 | 0 | 0 | 0 | 0 | 1 | 2.2 | 11 | 2.9 |  |
| CYP2C19 | *2\|*3 | PM | 3 | 0 | 3 | 0 | 0 | 0 | 0 | 0 | 0 | 0 | 0 | 0 | 0 | 0 |  |
| CYP2C19 | *2\|*35 | PM | 1 | 0 | 0 | 0 | 0 | 0 | 1 | 1.8 | 0 | 0 | 0 | 0 | 0 | 0 |  |
| CYP2C19 | *2\|*39 | IND | 1 | 0 | 1 | 0 | 0 | 0 | 0 | 0 | 0 | 0 | 0 | 0 | 0 | 0 |  |
| CYP2C19 | *2\|*4 | PM | 7 | 0.1 | 7 | 0.1 | 0 | 0 | 0 | 0 | 0 | 0 | 0 | 0 | 0 | 0 |  |
| CYP2C19 | *2\|*6 | PM | 2 | 0 | 2 | 0 | 0 | 0 | 0 | 0 | 0 | 0 | 0 | 0 | 0 | 0 |  |
| CYP2C19 | *2\|*8 | PM | 18 | 0.1 | 16 | 0.1 | 0 | 0 | 0 | 0 | 0 | 0 | 0 | 0 | 2 | 0.5 |  |
| CYP2C19 | *4\|*4 | PM | 1 | 0 | 1 | 0 | 0 | 0 | 0 | 0 | 0 | 0 | 0 | 0 | 0 | 0 |  |
| CYP2C9 | *1\|*1 | NM | 8647 | 63.3 | 8262 | 63 | 49 | 81.7 | 44 | 78.6 | 5 | 62.5 | 33 | 71.7 | 254 | 66.7 |  |
| CYP2C9 | *11\|*2 | IM | 5 | 0 | 4 | 0 | 0 | 0 | 0 | 0 | 0 | 0 | 0 | 0 | 1 | 0.3 |  |
| CYP2C9 | *11\|*3 | PM | 4 | 0 | 3 | 0 | 0 | 0 | 0 | 0 | 0 | 0 | 0 | 0 | 1 | 0.3 |  |
| CYP2C9 | *12\|*2 | IM | 4 | 0 | 4 | 0 | 0 | 0 | 0 | 0 | 0 | 0 | 0 | 0 | 0 | 0 |  |
| CYP2C9 | *12\|*3 | PM | 1 | 0 | 1 | 0 | 0 | 0 | 0 | 0 | 0 | 0 | 0 | 0 | 0 | 0 |  |
| CYP2C9 | *1\|*11 | IM | 64 | 0.5 | 61 | 0.5 | 0 | 0 | 1 | 1.8 | 0 | 0 | 1 | 2.2 | 1 | 0.3 |  |
| CYP2C9 | *1\|*12 | IM | 42 | 0.3 | 42 | 0.3 | 0 | 0 | 0 | 0 | 0 | 0 | 0 | 0 | 0 | 0 |  |
| CYP2C9 | *1\|*14 | IM | 3 | 0 | 2 | 0 | 0 | 0 | 0 | 0 | 0 | 0 | 0 | 0 | 1 | 0.3 |  |
| CYP2C9 | *1\|*16 | IM | 1 | 0 | 1 | 0 | 0 | 0 | 0 | 0 | 0 | 0 | 0 | 0 | 0 | 0 |  |
| CYP2C9 | *1\|*2 | IM | 2950 | 21.6 | 2847 | 21.7 | 3 | 5 | 8 | 14.3 | 0 | 0 | 8 | 17.4 | 84 | 22 |  |
| CYP2C9 | *1\|*29 | IM | 4 | 0 | 4 | 0 | 0 | 0 | 0 | 0 | 0 | 0 | 0 | 0 | 0 | 0 |  |
| CYP2C9 | *1\|*3 | IM | 1432 | 10.5 | 1384 | 10.5 | 8 | 13.3 | 2 | 3.6 | 2 | 25 | 3 | 6.5 | 33 | 8.7 |  |
| CYP2C9 | *1\|*5 | IM | 1 | 0 | 0 | 0 | 0 | 0 | 0 | 0 | 0 | 0 | 1 | 2.2 | 0 | 0 |  |
| CYP2C9 | *1\|*6 | IM | 2 | 0 | 2 | 0 | 0 | 0 | 0 | 0 | 0 | 0 | 0 | 0 | 0 | 0 |  |
| CYP2C9 | *1\|*8 | IM | 13 | 0.1 | 13 | 0.1 | 0 | 0 | 0 | 0 | 0 | 0 | 0 | 0 | 0 | 0 |  |
| CYP2C9 | *1\|*82 | IND | 4 | 0 | 4 | 0 | 0 | 0 | 0 | 0 | 0 | 0 | 0 | 0 | 0 | 0 |  |
| CYP2C9 | *1\|*9 | NM | 4 | 0 | 3 | 0 | 0 | 0 | 0 | 0 | 1 | 12.5 | 0 | 0 | 0 | 0 |  |
| CYP2C9 | *2\|*2 | IM | 237 | 1.7 | 234 | 1.8 | 0 | 0 | 1 | 1.8 | 0 | 0 | 0 | 0 | 2 | 0.5 |  |
| CYP2C9 | *2\|*3 | PM | 203 | 1.5 | 201 | 1.5 | 0 | 0 | 0 | 0 | 0 | 0 | 0 | 0 | 2 | 0.5 |  |
| CYP2C9 | *2\|*5 | IM | 1 | 0 | 1 | 0 | 0 | 0 | 0 | 0 | 0 | 0 | 0 | 0 | 0 | 0 |  |
| CYP2C9 | *2\|*6 | PM | 1 | 0 | 1 | 0 | 0 | 0 | 0 | 0 | 0 | 0 | 0 | 0 | 0 | 0 |  |
| CYP2C9 | *2\|*8 | IM | 3 | 0 | 3 | 0 | 0 | 0 | 0 | 0 | 0 | 0 | 0 | 0 | 0 | 0 |  |
| CYP2C9 | *3\|*3 | PM | 41 | 0.3 | 39 | 0.3 | 0 | 0 | 0 | 0 | 0 | 0 | 0 | 0 | 2 | 0.5 |  |
| CYP2C9 | *3\|*8 | PM | 3 | 0 | 3 | 0 | 0 | 0 | 0 | 0 | 0 | 0 | 0 | 0 | 0 | 0 |  |
| CYP2D6 | *1\|*1 | NM | 2306 | 16.9 | 2209 | 16.8 | 12 | 20 | 15 | 26.8 | 3 | 37.5 | 5 | 10.9 | 62 | 16.3 |  |
| CYP2D6 | *106\|*4 | IND | 1 | 0 | 1 | 0 | 0 | 0 | 0 | 0 | 0 | 0 | 0 | 0 | 0 | 0 |  |
| CYP2D6 | *10\|*10 | IM | 12 | 0.1 | 6 | 0 | 1 | 1.7 | 0 | 0 | 1 | 12.5 | 1 | 2.2 | 3 | 0.8 |  |
| CYP2D6 | *10\|*11 | IM | 1 | 0 | 1 | 0 | 0 | 0 | 0 | 0 | 0 | 0 | 0 | 0 | 0 | 0 |  |
| CYP2D6 | *10\|*17 | IM | 2 | 0 | 1 | 0 | 0 | 0 | 0 | 0 | 0 | 0 | 0 | 0 | 1 | 0.3 |  |
| CYP2D6 | *10\|*2 | NM | 54 | 0.4 | 48 | 0.4 | 2 | 3.3 | 0 | 0 | 0 | 0 | 1 | 2.2 | 3 | 0.8 |  |
| CYP2D6 | *10\|*33 | NM | 2 | 0 | 1 | 0 | 0 | 0 | 0 | 0 | 0 | 0 | 0 | 0 | 1 | 0.3 |  |
| CYP2D6 | *10\|*35 | NM | 20 | 0.1 | 19 | 0.1 | 0 | 0 | 0 | 0 | 0 | 0 | 0 | 0 | 1 | 0.3 |  |
| CYP2D6 | *10\|*4 | IM | 122 | 0.9 | 114 | 0.9 | 2 | 3.3 | 0 | 0 | 0 | 0 | 1 | 2.2 | 5 | 1.3 |  |
| CYP2D6 | *10\|*41 | IM | 32 | 0.2 | 30 | 0.2 | 1 | 1.7 | 0 | 0 | 0 | 0 | 1 | 2.2 | 0 | 0 |  |
| CYP2D6 | *10\|*45 | NM | 1 | 0 | 0 | 0 | 0 | 0 | 0 | 0 | 0 | 0 | 0 | 0 | 1 | 0.3 |  |
| CYP2D6 | *10\|*59 | IM | 1 | 0 | 1 | 0 | 0 | 0 | 0 | 0 | 0 | 0 | 0 | 0 | 0 | 0 |  |
| CYP2D6 | *10\|*6 | IM | 2 | 0 | 2 | 0 | 0 | 0 | 0 | 0 | 0 | 0 | 0 | 0 | 0 | 0 |  |
| CYP2D6 | *10\|*9 | IM | 7 | 0.1 | 7 | 0.1 | 0 | 0 | 0 | 0 | 0 | 0 | 0 | 0 | 0 | 0 |  |
| CYP2D6 | *116\|*4 | IND | 1 | 0 | 1 | 0 | 0 | 0 | 0 | 0 | 0 | 0 | 0 | 0 | 0 | 0 |  |
| CYP2D6 | *117\|*2 | IND | 3 | 0 | 3 | 0 | 0 | 0 | 0 | 0 | 0 | 0 | 0 | 0 | 0 | 0 |  |
| CYP2D6 | *117\|*35 | IND | 2 | 0 | 2 | 0 | 0 | 0 | 0 | 0 | 0 | 0 | 0 | 0 | 0 | 0 |  |
| CYP2D6 | *117\|*4 | IND | 8 | 0.1 | 8 | 0.1 | 0 | 0 | 0 | 0 | 0 | 0 | 0 | 0 | 0 | 0 |  |
| CYP2D6 | *117\|*6 | IND | 1 | 0 | 1 | 0 | 0 | 0 | 0 | 0 | 0 | 0 | 0 | 0 | 0 | 0 |  |
| CYP2D6 | *117\|*9 | IND | 2 | 0 | 2 | 0 | 0 | 0 | 0 | 0 | 0 | 0 | 0 | 0 | 0 | 0 |  |
| CYP2D6 | *11\|*2 | IM | 2 | 0 | 2 | 0 | 0 | 0 | 0 | 0 | 0 | 0 | 0 | 0 | 0 | 0 |  |
| CYP2D6 | *124\|*4 | PM | 1 | 0 | 1 | 0 | 0 | 0 | 0 | 0 | 0 | 0 | 0 | 0 | 0 | 0 |  |
| CYP2D6 | *140\|*4 | IND | 1 | 0 | 1 | 0 | 0 | 0 | 0 | 0 | 0 | 0 | 0 | 0 | 0 | 0 |  |
| CYP2D6 | *160\|*2 | IND | 3 | 0 | 3 | 0 | 0 | 0 | 0 | 0 | 0 | 0 | 0 | 0 | 0 | 0 |  |
| CYP2D6 | *160\|*4 | IND | 7 | 0.1 | 6 | 0 | 0 | 0 | 0 | 0 | 0 | 0 | 0 | 0 | 1 | 0.3 |  |
| CYP2D6 | *160\|*41 | IND | 1 | 0 | 1 | 0 | 0 | 0 | 0 | 0 | 0 | 0 | 0 | 0 | 0 | 0 |  |
| CYP2D6 | *17\|*17 | IM | 1 | 0 | 1 | 0 | 0 | 0 | 0 | 0 | 0 | 0 | 0 | 0 | 0 | 0 |  |
| CYP2D6 | *17\|*2 | NM | 2 | 0 | 2 | 0 | 0 | 0 | 0 | 0 | 0 | 0 | 0 | 0 | 0 | 0 |  |
| CYP2D6 | *17\|*3 | IM | 1 | 0 | 0 | 0 | 0 | 0 | 0 | 0 | 0 | 0 | 0 | 0 | 1 | 0.3 |  |
| CYP2D6 | *17\|*4 | IM | 9 | 0.1 | 8 | 0.1 | 0 | 0 | 1 | 1.8 | 0 | 0 | 0 | 0 | 0 | 0 |  |
| CYP2D6 | *17\|*41 | IM | 1 | 0 | 1 | 0 | 0 | 0 | 0 | 0 | 0 | 0 | 0 | 0 | 0 | 0 |  |
| CYP2D6 | *17\|*6 | IM | 2 | 0 | 2 | 0 | 0 | 0 | 0 | 0 | 0 | 0 | 0 | 0 | 0 | 0 |  |
| CYP2D6 | *1\|*10 | NM | 95 | 0.7 | 88 | 0.7 | 1 | 1.7 | 0 | 0 | 0 | 0 | 2 | 4.3 | 4 | 1 |  |
| CYP2D6 | *1\|*106 | IND | 1 | 0 | 1 | 0 | 0 | 0 | 0 | 0 | 0 | 0 | 0 | 0 | 0 | 0 |  |
| CYP2D6 | *1\|*11 | IM | 2 | 0 | 2 | 0 | 0 | 0 | 0 | 0 | 0 | 0 | 0 | 0 | 0 | 0 |  |
| CYP2D6 | *1\|*115 | IND | 1 | 0 | 1 | 0 | 0 | 0 | 0 | 0 | 0 | 0 | 0 | 0 | 0 | 0 |  |
| CYP2D6 | *1\|*116 | IND | 2 | 0 | 1 | 0 | 0 | 0 | 1 | 1.8 | 0 | 0 | 0 | 0 | 0 | 0 |  |
| CYP2D6 | *1\|*117 | IND | 9 | 0.1 | 9 | 0.1 | 0 | 0 | 0 | 0 | 0 | 0 | 0 | 0 | 0 | 0 |  |
| CYP2D6 | *1\|*124 | IM | 1 | 0 | 1 | 0 | 0 | 0 | 0 | 0 | 0 | 0 | 0 | 0 | 0 | 0 |  |
| CYP2D6 | *1\|*146 | IND | 1 | 0 | 1 | 0 | 0 | 0 | 0 | 0 | 0 | 0 | 0 | 0 | 0 | 0 |  |
| CYP2D6 | *1\|*17 | NM | 9 | 0.1 | 8 | 0.1 | 0 | 0 | 0 | 0 | 0 | 0 | 0 | 0 | 1 | 0.3 |  |
| CYP2D6 | *1\|*2 | NM | 1874 | 13.7 | 1792 | 13.7 | 12 | 20 | 14 | 25 | 0 | 0 | 9 | 19.6 | 47 | 12.3 |  |
| CYP2D6 | *1\|*21 | IM | 1 | 0 | 1 | 0 | 0 | 0 | 0 | 0 | 0 | 0 | 0 | 0 | 0 | 0 |  |
| CYP2D6 | *1\|*22 | IND | 10 | 0.1 | 10 | 0.1 | 0 | 0 | 0 | 0 | 0 | 0 | 0 | 0 | 0 | 0 |  |
| CYP2D6 | *1\|*28 | IND | 19 | 0.1 | 18 | 0.1 | 0 | 0 | 0 | 0 | 0 | 0 | 0 | 0 | 1 | 0.3 |  |
| CYP2D6 | *1\|*29 | NM | 2 | 0 | 2 | 0 | 0 | 0 | 0 | 0 | 0 | 0 | 0 | 0 | 0 | 0 |  |
| CYP2D6 | *1\|*3 | IM | 117 | 0.9 | 114 | 0.9 | 0 | 0 | 0 | 0 | 0 | 0 | 2 | 4.3 | 1 | 0.3 |  |
| CYP2D6 | *1\|*31 | IM | 1 | 0 | 1 | 0 | 0 | 0 | 0 | 0 | 0 | 0 | 0 | 0 | 0 | 0 |  |
| CYP2D6 | *1\|*33 | NM | 123 | 0.9 | 119 | 0.9 | 0 | 0 | 0 | 0 | 0 | 0 | 0 | 0 | 4 | 1 |  |
| CYP2D6 | *1\|*35 | NM | 284 | 2.1 | 273 | 2.1 | 0 | 0 | 1 | 1.8 | 0 | 0 | 2 | 4.3 | 8 | 2.1 |  |
| CYP2D6 | *1\|*39 | NM | 56 | 0.4 | 50 | 0.4 | 2 | 3.3 | 1 | 1.8 | 0 | 0 | 3 | 6.5 | 0 | 0 |  |
| CYP2D6 | *1\|*4 | IM | 2088 | 15.3 | 2017 | 15.4 | 5 | 8.3 | 4 | 7.1 | 0 | 0 | 3 | 6.5 | 59 | 15.5 |  |
| CYP2D6 | *1\|*41 | NM | 1002 | 7.3 | 966 | 7.4 | 5 | 8.3 | 2 | 3.6 | 1 | 12.5 | 1 | 2.2 | 27 | 7.1 |  |
| CYP2D6 | *1\|*45 | NM | 1 | 0 | 0 | 0 | 0 | 0 | 1 | 1.8 | 0 | 0 | 0 | 0 | 0 | 0 |  |
| CYP2D6 | *1\|*59 | NM | 43 | 0.3 | 41 | 0.3 | 0 | 0 | 0 | 0 | 0 | 0 | 0 | 0 | 2 | 0.5 |  |
| CYP2D6 | *1\|*6 | IM | 134 | 1 | 128 | 1 | 0 | 0 | 0 | 0 | 0 | 0 | 0 | 0 | 6 | 1.6 |  |
| CYP2D6 | *1\|*74 | IND | 19 | 0.1 | 16 | 0.1 | 0 | 0 | 2 | 3.6 | 0 | 0 | 0 | 0 | 1 | 0.3 |  |
| CYP2D6 | *1\|*75 | IND | 1 | 0 | 1 | 0 | 0 | 0 | 0 | 0 | 0 | 0 | 0 | 0 | 0 | 0 |  |
| CYP2D6 | *1\|*89 | IND | 1 | 0 | 1 | 0 | 0 | 0 | 0 | 0 | 0 | 0 | 0 | 0 | 0 | 0 |  |
| CYP2D6 | *1\|*9 | NM | 212 | 1.6 | 206 | 1.6 | 0 | 0 | 1 | 1.8 | 0 | 0 | 1 | 2.2 | 4 | 1 |  |
| CYP2D6 | *21\|*41 | IM | 1 | 0 | 1 | 0 | 0 | 0 | 0 | 0 | 0 | 0 | 0 | 0 | 0 | 0 |  |
| CYP2D6 | *22\|*3 | IND | 3 | 0 | 3 | 0 | 0 | 0 | 0 | 0 | 0 | 0 | 0 | 0 | 0 | 0 |  |
| CYP2D6 | *22\|*4 | IND | 8 | 0.1 | 8 | 0.1 | 0 | 0 | 0 | 0 | 0 | 0 | 0 | 0 | 0 | 0 |  |
| CYP2D6 | *22\|*41 | IND | 2 | 0 | 2 | 0 | 0 | 0 | 0 | 0 | 0 | 0 | 0 | 0 | 0 | 0 |  |
| CYP2D6 | *23\|*41 | IND | 1 | 0 | 1 | 0 | 0 | 0 | 0 | 0 | 0 | 0 | 0 | 0 | 0 | 0 |  |
| CYP2D6 | *28\|*28 | IND | 1 | 0 | 1 | 0 | 0 | 0 | 0 | 0 | 0 | 0 | 0 | 0 | 0 | 0 |  |
| CYP2D6 | *28\|*33 | IND | 2 | 0 | 2 | 0 | 0 | 0 | 0 | 0 | 0 | 0 | 0 | 0 | 0 | 0 |  |
| CYP2D6 | *28\|*35 | IND | 3 | 0 | 3 | 0 | 0 | 0 | 0 | 0 | 0 | 0 | 0 | 0 | 0 | 0 |  |
| CYP2D6 | *28\|*4 | IND | 23 | 0.2 | 23 | 0.2 | 0 | 0 | 0 | 0 | 0 | 0 | 0 | 0 | 0 | 0 |  |
| CYP2D6 | *28\|*41 | IND | 10 | 0.1 | 8 | 0.1 | 0 | 0 | 0 | 0 | 0 | 0 | 0 | 0 | 2 | 0.5 |  |
| CYP2D6 | *28\|*6 | IND | 2 | 0 | 2 | 0 | 0 | 0 | 0 | 0 | 0 | 0 | 0 | 0 | 0 | 0 |  |
| CYP2D6 | *28\|*9 | IND | 3 | 0 | 3 | 0 | 0 | 0 | 0 | 0 | 0 | 0 | 0 | 0 | 0 | 0 |  |
| CYP2D6 | *29\|*35 | NM | 1 | 0 | 1 | 0 | 0 | 0 | 0 | 0 | 0 | 0 | 0 | 0 | 0 | 0 |  |
| CYP2D6 | *29\|*4 | IM | 2 | 0 | 2 | 0 | 0 | 0 | 0 | 0 | 0 | 0 | 0 | 0 | 0 | 0 |  |
| CYP2D6 | *29\|*41 | IM | 1 | 0 | 1 | 0 | 0 | 0 | 0 | 0 | 0 | 0 | 0 | 0 | 0 | 0 |  |
| CYP2D6 | *2\|*2 | NM | 492 | 3.6 | 471 | 3.6 | 4 | 6.7 | 4 | 7.1 | 1 | 12.5 | 1 | 2.2 | 11 | 2.9 |  |
| CYP2D6 | *2\|*22 | IND | 3 | 0 | 3 | 0 | 0 | 0 | 0 | 0 | 0 | 0 | 0 | 0 | 0 | 0 |  |
| CYP2D6 | *2\|*28 | IND | 23 | 0.2 | 23 | 0.2 | 0 | 0 | 0 | 0 | 0 | 0 | 0 | 0 | 0 | 0 |  |
| CYP2D6 | *2\|*29 | NM | 2 | 0 | 2 | 0 | 0 | 0 | 0 | 0 | 0 | 0 | 0 | 0 | 0 | 0 |  |
| CYP2D6 | *2\|*3 | IM | 37 | 0.3 | 35 | 0.3 | 0 | 0 | 1 | 1.8 | 0 | 0 | 0 | 0 | 1 | 0.3 |  |
| CYP2D6 | *2\|*33 | NM | 65 | 0.5 | 64 | 0.5 | 0 | 0 | 0 | 0 | 0 | 0 | 0 | 0 | 1 | 0.3 |  |
| CYP2D6 | *2\|*34 | NM | 13 | 0.1 | 12 | 0.1 | 0 | 0 | 1 | 1.8 | 0 | 0 | 0 | 0 | 0 | 0 |  |
| CYP2D6 | *2\|*35 | NM | 271 | 2 | 253 | 1.9 | 0 | 0 | 2 | 3.6 | 0 | 0 | 0 | 0 | 16 | 4.2 |  |
| CYP2D6 | *2\|*39 | NM | 9 | 0.1 | 9 | 0.1 | 0 | 0 | 0 | 0 | 0 | 0 | 0 | 0 | 0 | 0 |  |
| CYP2D6 | *2\|*4 | IM | 858 | 6.3 | 829 | 6.3 | 2 | 3.3 | 2 | 3.6 | 0 | 0 | 2 | 4.3 | 23 | 6 |  |
| CYP2D6 | *2\|*41 | NM | 415 | 3 | 398 | 3 | 6 | 10 | 0 | 0 | 0 | 0 | 5 | 10.9 | 6 | 1.6 |  |
| CYP2D6 | *2\|*59 | NM | 30 | 0.2 | 30 | 0.2 | 0 | 0 | 0 | 0 | 0 | 0 | 0 | 0 | 0 | 0 |  |
| CYP2D6 | *2\|*6 | IM | 40 | 0.3 | 38 | 0.3 | 0 | 0 | 0 | 0 | 0 | 0 | 0 | 0 | 2 | 0.5 |  |
| CYP2D6 | *2\|*71 | IND | 1 | 0 | 0 | 0 | 0 | 0 | 0 | 0 | 0 | 0 | 1 | 2.2 | 0 | 0 |  |
| CYP2D6 | *2\|*74 | IND | 2 | 0 | 2 | 0 | 0 | 0 | 0 | 0 | 0 | 0 | 0 | 0 | 0 | 0 |  |
| CYP2D6 | *2\|*9 | NM | 88 | 0.6 | 86 | 0.7 | 0 | 0 | 1 | 1.8 | 0 | 0 | 0 | 0 | 1 | 0.3 |  |
| CYP2D6 | *33\|*33 | NM | 7 | 0.1 | 7 | 0.1 | 0 | 0 | 0 | 0 | 0 | 0 | 0 | 0 | 0 | 0 |  |
| CYP2D6 | *33\|*35 | NM | 10 | 0.1 | 10 | 0.1 | 0 | 0 | 0 | 0 | 0 | 0 | 0 | 0 | 0 | 0 |  |
| CYP2D6 | *33\|*39 | NM | 1 | 0 | 1 | 0 | 0 | 0 | 0 | 0 | 0 | 0 | 0 | 0 | 0 | 0 |  |
| CYP2D6 | *33\|*4 | IM | 53 | 0.4 | 51 | 0.4 | 0 | 0 | 0 | 0 | 0 | 0 | 0 | 0 | 2 | 0.5 |  |
| CYP2D6 | *33\|*41 | NM | 35 | 0.3 | 33 | 0.3 | 0 | 0 | 0 | 0 | 0 | 0 | 0 | 0 | 2 | 0.5 |  |
| CYP2D6 | *33\|*59 | NM | 1 | 0 | 1 | 0 | 0 | 0 | 0 | 0 | 0 | 0 | 0 | 0 | 0 | 0 |  |
| CYP2D6 | *33\|*6 | IM | 1 | 0 | 1 | 0 | 0 | 0 | 0 | 0 | 0 | 0 | 0 | 0 | 0 | 0 |  |
| CYP2D6 | *33\|*9 | NM | 3 | 0 | 3 | 0 | 0 | 0 | 0 | 0 | 0 | 0 | 0 | 0 | 0 | 0 |  |
| CYP2D6 | *34\|*35 | NM | 1 | 0 | 1 | 0 | 0 | 0 | 0 | 0 | 0 | 0 | 0 | 0 | 0 | 0 |  |
| CYP2D6 | *35\|*35 | NM | 52 | 0.4 | 49 | 0.4 | 0 | 0 | 0 | 0 | 0 | 0 | 0 | 0 | 3 | 0.8 |  |
| CYP2D6 | *35\|*39 | NM | 7 | 0.1 | 7 | 0.1 | 0 | 0 | 0 | 0 | 0 | 0 | 0 | 0 | 0 | 0 |  |
| CYP2D6 | *35\|*4 | IM | 300 | 2.2 | 289 | 2.2 | 0 | 0 | 1 | 1.8 | 0 | 0 | 0 | 0 | 10 | 2.6 |  |
| CYP2D6 | *35\|*41 | NM | 127 | 0.9 | 125 | 1 | 0 | 0 | 0 | 0 | 0 | 0 | 0 | 0 | 2 | 0.5 |  |
| CYP2D6 | *35\|*43 | NM | 1 | 0 | 1 | 0 | 0 | 0 | 0 | 0 | 0 | 0 | 0 | 0 | 0 | 0 |  |
| CYP2D6 | *35\|*59 | NM | 10 | 0.1 | 10 | 0.1 | 0 | 0 | 0 | 0 | 0 | 0 | 0 | 0 | 0 | 0 |  |
| CYP2D6 | *35\|*6 | IM | 6 | 0 | 6 | 0 | 0 | 0 | 0 | 0 | 0 | 0 | 0 | 0 | 0 | 0 |  |
| CYP2D6 | *35\|*74 | IND | 1 | 0 | 1 | 0 | 0 | 0 | 0 | 0 | 0 | 0 | 0 | 0 | 0 | 0 |  |
| CYP2D6 | *35\|*9 | NM | 34 | 0.2 | 33 | 0.3 | 0 | 0 | 0 | 0 | 0 | 0 | 0 | 0 | 1 | 0.3 |  |
| CYP2D6 | *39\|*4 | IM | 22 | 0.2 | 22 | 0.2 | 0 | 0 | 0 | 0 | 0 | 0 | 0 | 0 | 0 | 0 |  |
| CYP2D6 | *39\|*41 | NM | 3 | 0 | 3 | 0 | 0 | 0 | 0 | 0 | 0 | 0 | 0 | 0 | 0 | 0 |  |
| CYP2D6 | *39\|*6 | IM | 1 | 0 | 1 | 0 | 0 | 0 | 0 | 0 | 0 | 0 | 0 | 0 | 0 | 0 |  |
| CYP2D6 | *39\|*65 | IND | 1 | 0 | 1 | 0 | 0 | 0 | 0 | 0 | 0 | 0 | 0 | 0 | 0 | 0 |  |
| CYP2D6 | *39\|*74 | IND | 21 | 0.2 | 21 | 0.2 | 0 | 0 | 0 | 0 | 0 | 0 | 0 | 0 | 0 | 0 |  |
| CYP2D6 | *3\|*33 | IM | 1 | 0 | 1 | 0 | 0 | 0 | 0 | 0 | 0 | 0 | 0 | 0 | 0 | 0 |  |
| CYP2D6 | *3\|*35 | IM | 7 | 0.1 | 7 | 0.1 | 0 | 0 | 0 | 0 | 0 | 0 | 0 | 0 | 0 | 0 |  |
| CYP2D6 | *3\|*39 | IM | 3 | 0 | 2 | 0 | 0 | 0 | 0 | 0 | 0 | 0 | 0 | 0 | 1 | 0.3 |  |
| CYP2D6 | *3\|*4 | PM | 83 | 0.6 | 82 | 0.6 | 0 | 0 | 0 | 0 | 0 | 0 | 0 | 0 | 1 | 0.3 |  |
| CYP2D6 | *3\|*41 | IM | 17 | 0.1 | 17 | 0.1 | 0 | 0 | 0 | 0 | 0 | 0 | 0 | 0 | 0 | 0 |  |
| CYP2D6 | *3\|*6 | PM | 2 | 0 | 2 | 0 | 0 | 0 | 0 | 0 | 0 | 0 | 0 | 0 | 0 | 0 |  |
| CYP2D6 | *3\|*9 | IM | 8 | 0.1 | 8 | 0.1 | 0 | 0 | 0 | 0 | 0 | 0 | 0 | 0 | 0 | 0 |  |
| CYP2D6 | *41\|*41 | IM | 195 | 1.4 | 191 | 1.5 | 1 | 1.7 | 0 | 0 | 0 | 0 | 0 | 0 | 3 | 0.8 |  |
| CYP2D6 | *41\|*59 | IM | 8 | 0.1 | 8 | 0.1 | 0 | 0 | 0 | 0 | 0 | 0 | 0 | 0 | 0 | 0 |  |
| CYP2D6 | *41\|*6 | IM | 21 | 0.2 | 20 | 0.2 | 0 | 0 | 0 | 0 | 0 | 0 | 0 | 0 | 1 | 0.3 |  |
| CYP2D6 | *41\|*9 | IM | 54 | 0.4 | 51 | 0.4 | 0 | 0 | 0 | 0 | 0 | 0 | 0 | 0 | 3 | 0.8 |  |
| CYP2D6 | *45\|*45 | NM | 2 | 0 | 1 | 0 | 0 | 0 | 0 | 0 | 0 | 0 | 0 | 0 | 1 | 0.3 |  |
| CYP2D6 | *4\|*4 | PM | 686 | 5 | 663 | 5.1 | 3 | 5 | 0 | 0 | 1 | 12.5 | 2 | 4.3 | 17 | 4.5 |  |
| CYP2D6 | *4\|*41 | IM | 535 | 3.9 | 510 | 3.9 | 1 | 1.7 | 0 | 0 | 1 | 12.5 | 3 | 6.5 | 20 | 5.2 |  |
| CYP2D6 | *4\|*45 | IM | 1 | 0 | 1 | 0 | 0 | 0 | 0 | 0 | 0 | 0 | 0 | 0 | 0 | 0 |  |
| CYP2D6 | *4\|*49 | IM | 1 | 0 | 1 | 0 | 0 | 0 | 0 | 0 | 0 | 0 | 0 | 0 | 0 | 0 |  |
| CYP2D6 | *4\|*59 | IM | 18 | 0.1 | 17 | 0.1 | 0 | 0 | 0 | 0 | 0 | 0 | 0 | 0 | 1 | 0.3 |  |
| CYP2D6 | *4\|*6 | PM | 11 | 0.1 | 11 | 0.1 | 0 | 0 | 0 | 0 | 0 | 0 | 0 | 0 | 0 | 0 |  |
| CYP2D6 | *4\|*65 | IND | 4 | 0 | 4 | 0 | 0 | 0 | 0 | 0 | 0 | 0 | 0 | 0 | 0 | 0 |  |
| CYP2D6 | *4\|*69 | PM | 2 | 0 | 2 | 0 | 0 | 0 | 0 | 0 | 0 | 0 | 0 | 0 | 0 | 0 |  |
| CYP2D6 | *4\|*7 | PM | 1 | 0 | 1 | 0 | 0 | 0 | 0 | 0 | 0 | 0 | 0 | 0 | 0 | 0 |  |
| CYP2D6 | *4\|*71 | IND | 1 | 0 | 0 | 0 | 0 | 0 | 0 | 0 | 0 | 0 | 0 | 0 | 1 | 0.3 |  |
| CYP2D6 | *4\|*74 | IND | 41 | 0.3 | 39 | 0.3 | 0 | 0 | 0 | 0 | 0 | 0 | 0 | 0 | 2 | 0.5 |  |
| CYP2D6 | *4\|*89 | IND | 1 | 0 | 1 | 0 | 0 | 0 | 0 | 0 | 0 | 0 | 0 | 0 | 0 | 0 |  |
| CYP2D6 | *4\|*9 | IM | 114 | 0.8 | 112 | 0.9 | 0 | 0 | 0 | 0 | 0 | 0 | 0 | 0 | 2 | 0.5 |  |
| CYP2D6 | *59\|*6 | IM | 2 | 0 | 2 | 0 | 0 | 0 | 0 | 0 | 0 | 0 | 0 | 0 | 0 | 0 |  |
| CYP2D6 | *59\|*65 | IND | 1 | 0 | 1 | 0 | 0 | 0 | 0 | 0 | 0 | 0 | 0 | 0 | 0 | 0 |  |
| CYP2D6 | *59\|*9 | IM | 2 | 0 | 2 | 0 | 0 | 0 | 0 | 0 | 0 | 0 | 0 | 0 | 0 | 0 |  |
| CYP2D6 | *6\|*6 | PM | 3 | 0 | 3 | 0 | 0 | 0 | 0 | 0 | 0 | 0 | 0 | 0 | 0 | 0 |  |
| CYP2D6 | *6\|*74 | IND | 1 | 0 | 1 | 0 | 0 | 0 | 0 | 0 | 0 | 0 | 0 | 0 | 0 | 0 |  |
| CYP2D6 | *6\|*9 | IM | 2 | 0 | 2 | 0 | 0 | 0 | 0 | 0 | 0 | 0 | 0 | 0 | 0 | 0 |  |
| CYP2D6 | *74\|*9 | IND | 1 | 0 | 0 | 0 | 0 | 0 | 1 | 1.8 | 0 | 0 | 0 | 0 | 0 | 0 |  |
| CYP2D6 | *9\|*9 | IM | 11 | 0.1 | 10 | 0.1 | 0 | 0 | 0 | 0 | 0 | 0 | 0 | 0 | 1 | 0.3 |  |
| CYP3A5 | *1\|*1 | NM | 60 | 0.4 | 49 | 0.4 | 4 | 6.7 | 5 | 8.9 | 1 | 12.5 | 0 | 0 | 1 | 0.3 |  |
| CYP3A5 | *1\|*3 | IM | 1627 | 11.9 | 1536 | 11.7 | 24 | 40 | 16 | 28.6 | 2 | 25 | 12 | 26.1 | 37 | 9.7 |  |
| CYP3A5 | *1\|*6 | IM | 2 | 0 | 1 | 0 | 0 | 0 | 1 | 1.8 | 0 | 0 | 0 | 0 | 0 | 0 |  |
| CYP3A5 | *3\|*3 | PM | 11967 | 87.5 | 11522 | 87.8 | 32 | 53.3 | 34 | 60.7 | 3 | 37.5 | 34 | 73.9 | 342 | 89.8 |  |
| CYP3A5 | *3\|*6 | PM | 11 | 0.1 | 9 | 0.1 | 0 | 0 | 0 | 0 | 1 | 12.5 | 0 | 0 | 1 | 0.3 |  |
| CYP3A5 | *3\|*7 | PM | 3 | 0 | 2 | 0 | 0 | 0 | 0 | 0 | 1 | 12.5 | 0 | 0 | 0 | 0 |  |
| DPYD | Reference\|Reference | NM | 3454 | 25.3 | 3323 | 25.3 | 19 | 31.7 | 12 | 21.4 | 3 | 37.5 | 10 | 21.7 | 87 | 22.8 |  |
| DPYD | *13\|*5 | IM | 7 | 0.1 | 7 | 0.1 | 0 | 0 | 0 | 0 | 0 | 0 | 0 | 0 | 0 | 0 |  |
| DPYD | *13\|*6 | IM | 2 | 0 | 2 | 0 | 0 | 0 | 0 | 0 | 0 | 0 | 0 | 0 | 0 | 0 |  |
| DPYD | *13\|*9A | IM | 1 | 0 | 1 | 0 | 0 | 0 | 0 | 0 | 0 | 0 | 0 | 0 | 0 | 0 |  |
| DPYD | *13\|Reference | IM | 13 | 0.1 | 13 | 0.1 | 0 | 0 | 0 | 0 | 0 | 0 | 0 | 0 | 0 | 0 |  |
| DPYD | *13\|c.1896T>C | IM | 2 | 0 | 2 | 0 | 0 | 0 | 0 | 0 | 0 | 0 | 0 | 0 | 0 | 0 |  |
| DPYD | *2A\|*2A | PM | 1 | 0 | 1 | 0 | 0 | 0 | 0 | 0 | 0 | 0 | 0 | 0 | 0 | 0 |  |
| DPYD | *2A\|*4 | IM | 1 | 0 | 1 | 0 | 0 | 0 | 0 | 0 | 0 | 0 | 0 | 0 | 0 | 0 |  |
| DPYD | *2A\|*5 | IM | 29 | 0.2 | 28 | 0.2 | 0 | 0 | 0 | 0 | 0 | 0 | 0 | 0 | 1 | 0.3 |  |
| DPYD | *2A\|*6 | IM | 7 | 0.1 | 7 | 0.1 | 0 | 0 | 0 | 0 | 0 | 0 | 0 | 0 | 0 | 0 |  |
| DPYD | *2A\|*9A | IM | 16 | 0.1 | 15 | 0.1 | 0 | 0 | 0 | 0 | 0 | 0 | 0 | 0 | 1 | 0.3 |  |
| DPYD | *2A\|HapB3 | PM | 3 | 0 | 3 | 0 | 0 | 0 | 0 | 0 | 0 | 0 | 0 | 0 | 0 | 0 |  |
| DPYD | *2A\|Reference | IM | 71 | 0.5 | 69 | 0.5 | 0 | 0 | 0 | 0 | 1 | 12.5 | 0 | 0 | 1 | 0.3 |  |
| DPYD | *2A\|c.2846A>T | PM | 2 | 0 | 2 | 0 | 0 | 0 | 0 | 0 | 0 | 0 | 0 | 0 | 0 | 0 |  |
| DPYD | *2A\|c.496A>G | IM | 2 | 0 | 1 | 0 | 1 | 1.7 | 0 | 0 | 0 | 0 | 0 | 0 | 0 | 0 |  |
| DPYD | *4\|*4 | NM | 1 | 0 | 1 | 0 | 0 | 0 | 0 | 0 | 0 | 0 | 0 | 0 | 0 | 0 |  |
| DPYD | *4\|*5 | NM | 118 | 0.9 | 107 | 0.8 | 0 | 0 | 0 | 0 | 0 | 0 | 4 | 8.7 | 7 | 1.8 |  |
| DPYD | *4\|*6 | NM | 25 | 0.2 | 24 | 0.2 | 0 | 0 | 0 | 0 | 0 | 0 | 0 | 0 | 1 | 0.3 |  |
| DPYD | *4\|*9A | NM | 96 | 0.7 | 94 | 0.7 | 0 | 0 | 0 | 0 | 0 | 0 | 0 | 0 | 2 | 0.5 |  |
| DPYD | *4\|HapB3 | IM | 11 | 0.1 | 11 | 0.1 | 0 | 0 | 0 | 0 | 0 | 0 | 0 | 0 | 0 | 0 |  |
| DPYD | *4\|Reference | NM | 343 | 2.5 | 338 | 2.6 | 0 | 0 | 1 | 1.8 | 0 | 0 | 1 | 2.2 | 3 | 0.8 |  |
| DPYD | *4\|c.1896T>C | NM | 15 | 0.1 | 15 | 0.1 | 0 | 0 | 0 | 0 | 0 | 0 | 0 | 0 | 0 | 0 |  |
| DPYD | *4\|c.2846A>T | IM | 5 | 0 | 5 | 0 | 0 | 0 | 0 | 0 | 0 | 0 | 0 | 0 | 0 | 0 |  |
| DPYD | *4\|c.496A>G | NM | 12 | 0.1 | 12 | 0.1 | 0 | 0 | 0 | 0 | 0 | 0 | 0 | 0 | 0 | 0 |  |
| DPYD | *4\|c.775A>G | NM | 1 | 0 | 1 | 0 | 0 | 0 | 0 | 0 | 0 | 0 | 0 | 0 | 0 | 0 |  |
| DPYD | *4\|c.934C>T | NM | 1 | 0 | 1 | 0 | 0 | 0 | 0 | 0 | 0 | 0 | 0 | 0 | 0 | 0 |  |
| DPYD | *5\|*5 | NM | 554 | 4.1 | 530 | 4 | 0 | 0 | 4 | 7.1 | 0 | 0 | 0 | 0 | 20 | 5.2 |  |
| DPYD | *5\|*6 | NM | 216 | 1.6 | 210 | 1.6 | 0 | 0 | 0 | 0 | 0 | 0 | 0 | 0 | 6 | 1.6 |  |
| DPYD | *5\|*7 | IM | 1 | 0 | 1 | 0 | 0 | 0 | 0 | 0 | 0 | 0 | 0 | 0 | 0 | 0 |  |
| DPYD | *5\|*9A | NM | 883 | 6.5 | 848 | 6.5 | 2 | 3.3 | 9 | 16.1 | 0 | 0 | 3 | 6.5 | 21 | 5.5 |  |
| DPYD | *5\|HapB3 | IM | 112 | 0.8 | 110 | 0.8 | 0 | 0 | 0 | 0 | 0 | 0 | 1 | 2.2 | 1 | 0.3 |  |
| DPYD | *5\|Reference | NM | 2756 | 20.2 | 2635 | 20.1 | 9 | 15 | 11 | 19.6 | 1 | 12.5 | 11 | 23.9 | 89 | 23.4 |  |
| DPYD | *5\|c.1218G>A | NM | 1 | 0 | 1 | 0 | 0 | 0 | 0 | 0 | 0 | 0 | 0 | 0 | 0 | 0 |  |
| DPYD | *5\|c.1278G>T | NM | 1 | 0 | 1 | 0 | 0 | 0 | 0 | 0 | 0 | 0 | 0 | 0 | 0 | 0 |  |
| DPYD | *5\|c.1896T>C | NM | 101 | 0.7 | 98 | 0.7 | 0 | 0 | 1 | 1.8 | 0 | 0 | 0 | 0 | 2 | 0.5 |  |
| DPYD | *5\|c.2482G>A | NM | 1 | 0 | 1 | 0 | 0 | 0 | 0 | 0 | 0 | 0 | 0 | 0 | 0 | 0 |  |
| DPYD | *5\|c.2846A>T | IM | 33 | 0.2 | 33 | 0.3 | 0 | 0 | 0 | 0 | 0 | 0 | 0 | 0 | 0 | 0 |  |
| DPYD | *5\|c.496A>G | NM | 104 | 0.8 | 98 | 0.7 | 0 | 0 | 1 | 1.8 | 0 | 0 | 1 | 2.2 | 4 | 1 |  |
| DPYD | *5\|c.775A>G | NM | 2 | 0 | 2 | 0 | 0 | 0 | 0 | 0 | 0 | 0 | 0 | 0 | 0 | 0 |  |
| DPYD | *6\|*6 | NM | 18 | 0.1 | 16 | 0.1 | 0 | 0 | 0 | 0 | 0 | 0 | 1 | 2.2 | 1 | 0.3 |  |
| DPYD | *6\|*9A | NM | 158 | 1.2 | 151 | 1.2 | 2 | 3.3 | 0 | 0 | 0 | 0 | 1 | 2.2 | 4 | 1 |  |
| DPYD | *6\|HapB3 | IM | 23 | 0.2 | 22 | 0.2 | 1 | 1.7 | 0 | 0 | 0 | 0 | 0 | 0 | 0 | 0 |  |
| DPYD | *6\|Reference | NM | 506 | 3.7 | 480 | 3.7 | 5 | 8.3 | 1 | 1.8 | 1 | 12.5 | 0 | 0 | 19 | 5 |  |
| DPYD | *6\|c.1896T>C | NM | 22 | 0.2 | 19 | 0.1 | 0 | 0 | 1 | 1.8 | 0 | 0 | 0 | 0 | 2 | 0.5 |  |
| DPYD | *6\|c.2846A>T | IM | 2 | 0 | 2 | 0 | 0 | 0 | 0 | 0 | 0 | 0 | 0 | 0 | 0 | 0 |  |
| DPYD | *6\|c.496A>G | NM | 21 | 0.2 | 20 | 0.2 | 0 | 0 | 0 | 0 | 0 | 0 | 0 | 0 | 1 | 0.3 |  |
| DPYD | *6\|c.775A>G | NM | 2 | 0 | 2 | 0 | 0 | 0 | 0 | 0 | 0 | 0 | 0 | 0 | 0 | 0 |  |
| DPYD | *7\|Reference | IM | 3 | 0 | 2 | 0 | 0 | 0 | 0 | 0 | 0 | 0 | 0 | 0 | 1 | 0.3 |  |
| DPYD | *9A\|*9A | NM | 359 | 2.6 | 344 | 2.6 | 1 | 1.7 | 0 | 0 | 1 | 12.5 | 2 | 4.3 | 11 | 2.9 |  |
| DPYD | *9A\|HapB3 | IM | 84 | 0.6 | 82 | 0.6 | 0 | 0 | 0 | 0 | 0 | 0 | 0 | 0 | 2 | 0.5 |  |
| DPYD | *9A\|Reference | NM | 2198 | 16.1 | 2107 | 16.1 | 16 | 26.7 | 7 | 12.5 | 0 | 0 | 7 | 15.2 | 61 | 16 |  |
| DPYD | *9A\|c.1218G>A | NM | 2 | 0 | 2 | 0 | 0 | 0 | 0 | 0 | 0 | 0 | 0 | 0 | 0 | 0 |  |
| DPYD | *9A\|c.1278G>T | NM | 1 | 0 | 1 | 0 | 0 | 0 | 0 | 0 | 0 | 0 | 0 | 0 | 0 | 0 |  |
| DPYD | *9A\|c.1896T>C | NM | 97 | 0.7 | 92 | 0.7 | 0 | 0 | 2 | 3.6 | 0 | 0 | 0 | 0 | 3 | 0.8 |  |
| DPYD | *9A\|c.2846A>T | IM | 33 | 0.2 | 33 | 0.3 | 0 | 0 | 0 | 0 | 0 | 0 | 0 | 0 | 0 | 0 |  |
| DPYD | *9A\|c.451A>G | NM | 1 | 0 | 0 | 0 | 0 | 0 | 1 | 1.8 | 0 | 0 | 0 | 0 | 0 | 0 |  |
| DPYD | *9A\|c.496A>G | NM | 87 | 0.6 | 85 | 0.6 | 0 | 0 | 0 | 0 | 0 | 0 | 0 | 0 | 2 | 0.5 |  |
| DPYD | *9A\|c.775A>G | NM | 5 | 0 | 5 | 0 | 0 | 0 | 0 | 0 | 0 | 0 | 0 | 0 | 0 | 0 |  |
| DPYD | HapB3\|HapB3 | IM | 7 | 0.1 | 7 | 0.1 | 0 | 0 | 0 | 0 | 0 | 0 | 0 | 0 | 0 | 0 |  |
| DPYD | HapB3\|Reference | IM | 278 | 2 | 269 | 2.1 | 1 | 1.7 | 0 | 0 | 0 | 0 | 0 | 0 | 8 | 2.1 |  |
| DPYD | c.1278G>T\|Reference | NM | 2 | 0 | 2 | 0 | 0 | 0 | 0 | 0 | 0 | 0 | 0 | 0 | 0 | 0 |  |
| DPYD | c.1371C>T\|Reference | NM | 1 | 0 | 0 | 0 | 0 | 0 | 0 | 0 | 0 | 0 | 1 | 2.2 | 0 | 0 |  |
| DPYD | c.1896T>C\|HapB3 | IM | 13 | 0.1 | 13 | 0.1 | 0 | 0 | 0 | 0 | 0 | 0 | 0 | 0 | 0 | 0 |  |
| DPYD | c.1896T>C\|Reference | NM | 288 | 2.1 | 268 | 2 | 3 | 5 | 4 | 7.1 | 1 | 12.5 | 3 | 6.5 | 9 | 2.4 |  |
| DPYD | c.1896T>C\|c.1896T>C | NM | 6 | 0 | 5 | 0 | 0 | 0 | 1 | 1.8 | 0 | 0 | 0 | 0 | 0 | 0 |  |
| DPYD | c.1896T>C\|c.2161G>A | NM | 1 | 0 | 1 | 0 | 0 | 0 | 0 | 0 | 0 | 0 | 0 | 0 | 0 | 0 |  |
| DPYD | c.1896T>C\|c.2846A>T | IM | 2 | 0 | 2 | 0 | 0 | 0 | 0 | 0 | 0 | 0 | 0 | 0 | 0 | 0 |  |
| DPYD | c.1896T>C\|c.496A>G | NM | 7 | 0.1 | 7 | 0.1 | 0 | 0 | 0 | 0 | 0 | 0 | 0 | 0 | 0 | 0 |  |
| DPYD | c.2161G>A\|Reference | NM | 2 | 0 | 2 | 0 | 0 | 0 | 0 | 0 | 0 | 0 | 0 | 0 | 0 | 0 |  |
| DPYD | c.2279C>T\|Reference | IM | 1 | 0 | 1 | 0 | 0 | 0 | 0 | 0 | 0 | 0 | 0 | 0 | 0 | 0 |  |
| DPYD | c.2846A>T\|HapB3 | IM | 4 | 0 | 4 | 0 | 0 | 0 | 0 | 0 | 0 | 0 | 0 | 0 | 0 | 0 |  |
| DPYD | c.2846A>T\|Reference | IM | 97 | 0.7 | 95 | 0.7 | 0 | 0 | 0 | 0 | 0 | 0 | 0 | 0 | 2 | 0.5 |  |
| DPYD | c.2846A>T\|c.2846A>T | IM | 2 | 0 | 2 | 0 | 0 | 0 | 0 | 0 | 0 | 0 | 0 | 0 | 0 | 0 |  |
| DPYD | c.2846A>T\|c.496A>G | IM | 6 | 0 | 6 | 0 | 0 | 0 | 0 | 0 | 0 | 0 | 0 | 0 | 0 | 0 |  |
| DPYD | c.2872A>G\|Reference | IM | 1 | 0 | 1 | 0 | 0 | 0 | 0 | 0 | 0 | 0 | 0 | 0 | 0 | 0 |  |
| DPYD | c.3061G>C\|Reference | NM | 1 | 0 | 1 | 0 | 0 | 0 | 0 | 0 | 0 | 0 | 0 | 0 | 0 | 0 |  |
| DPYD | c.496A>G\|HapB3 | IM | 17 | 0.1 | 16 | 0.1 | 0 | 0 | 0 | 0 | 0 | 0 | 0 | 0 | 1 | 0.3 |  |
| DPYD | c.496A>G\|Reference | NM | 294 | 2.2 | 286 | 2.2 | 0 | 0 | 0 | 0 | 0 | 0 | 0 | 0 | 8 | 2.1 |  |
| DPYD | c.496A>G\|c.496A>G | NM | 6 | 0 | 6 | 0 | 0 | 0 | 0 | 0 | 0 | 0 | 0 | 0 | 0 | 0 |  |
| DPYD | c.557A>G\|Reference | IM | 2 | 0 | 2 | 0 | 0 | 0 | 0 | 0 | 0 | 0 | 0 | 0 | 0 | 0 |  |
| DPYD | c.601A>C\|Reference | IM | 1 | 0 | 1 | 0 | 0 | 0 | 0 | 0 | 0 | 0 | 0 | 0 | 0 | 0 |  |
| DPYD | c.775A>G\|Reference | NM | 4 | 0 | 4 | 0 | 0 | 0 | 0 | 0 | 0 | 0 | 0 | 0 | 0 | 0 |  |
| DPYD | c.934C>T\|Reference | NM | 1 | 0 | 1 | 0 | 0 | 0 | 0 | 0 | 0 | 0 | 0 | 0 | 0 | 0 |  |
| NUDT15 | *1\|*1 | NM | 13358 | 97.7 | 12838 | 97.9 | 49 | 81.7 | 47 | 83.9 | 8 | 100 | 42 | 91.3 | 374 | 98.2 |  |
| NUDT15 | *1\|*12 | IND | 8 | 0.1 | 8 | 0.1 | 0 | 0 | 0 | 0 | 0 | 0 | 0 | 0 | 0 | 0 |  |
| NUDT15 | *1\|*14 | IND | 6 | 0 | 5 | 0 | 0 | 0 | 0 | 0 | 0 | 0 | 0 | 0 | 1 | 0.3 |  |
| NUDT15 | *1\|*2 | IM | 59 | 0.4 | 49 | 0.4 | 2 | 3.3 | 7 | 12.5 | 0 | 0 | 0 | 0 | 1 | 0.3 |  |
| NUDT15 | *1\|*3 | IM | 130 | 1 | 113 | 0.9 | 9 | 15 | 2 | 3.6 | 0 | 0 | 4 | 8.7 | 2 | 0.5 |  |
| NUDT15 | *1\|*5 | IND | 1 | 0 | 1 | 0 | 0 | 0 | 0 | 0 | 0 | 0 | 0 | 0 | 0 | 0 |  |
| NUDT15 | *1\|*6 | IND | 57 | 0.4 | 55 | 0.4 | 0 | 0 | 0 | 0 | 0 | 0 | 0 | 0 | 2 | 0.5 |  |
| NUDT15 | *1\|*9 | IM | 50 | 0.4 | 49 | 0.4 | 0 | 0 | 0 | 0 | 0 | 0 | 0 | 0 | 1 | 0.3 |  |
| NUDT15 | *3\|*6 | IM | 1 | 0 | 1 | 0 | 0 | 0 | 0 | 0 | 0 | 0 | 0 | 0 | 0 | 0 |  |
| SLCO1B1 | *1\|*1 | Normal | 4430 | 32.4 | 4252 | 32.4 | 15 | 25 | 15 | 26.8 | 0 | 0 | 6 | 13 | 142 | 37.3 |  |
| SLCO1B1 | *14\|*14 | Increased | 328 | 2.4 | 316 | 2.4 | 0 | 0 | 2 | 3.6 | 0 | 0 | 2 | 4.3 | 8 | 2.1 |  |
| SLCO1B1 | *14\|*15 | Decreased | 508 | 3.7 | 491 | 3.7 | 0 | 0 | 0 | 0 | 0 | 0 | 1 | 2.2 | 16 | 4.2 |  |
| SLCO1B1 | *14\|*19 | IND | 6 | 0 | 6 | 0 | 0 | 0 | 0 | 0 | 0 | 0 | 0 | 0 | 0 | 0 |  |
| SLCO1B1 | *14\|*20 | Increased | 218 | 1.6 | 211 | 1.6 | 2 | 3.3 | 0 | 0 | 0 | 0 | 0 | 0 | 5 | 1.3 |  |
| SLCO1B1 | *14\|*27 | IND | 2 | 0 | 2 | 0 | 0 | 0 | 0 | 0 | 0 | 0 | 0 | 0 | 0 | 0 |  |
| SLCO1B1 | *14\|*28 | IND | 1 | 0 | 1 | 0 | 0 | 0 | 0 | 0 | 0 | 0 | 0 | 0 | 0 | 0 |  |
| SLCO1B1 | *14\|*30 | IND | 1 | 0 | 1 | 0 | 0 | 0 | 0 | 0 | 0 | 0 | 0 | 0 | 0 | 0 |  |
| SLCO1B1 | *14\|*37 | Normal | 252 | 1.8 | 235 | 1.8 | 3 | 5 | 3 | 5.4 | 1 | 12.5 | 2 | 4.3 | 8 | 2.1 |  |
| SLCO1B1 | *14\|*40 | IND | 1 | 0 | 1 | 0 | 0 | 0 | 0 | 0 | 0 | 0 | 0 | 0 | 0 | 0 |  |
| SLCO1B1 | *14\|*5 | Decreased | 138 | 1 | 136 | 1 | 0 | 0 | 0 | 0 | 0 | 0 | 0 | 0 | 2 | 0.5 |  |
| SLCO1B1 | *15\|*15 | Poor | 188 | 1.4 | 182 | 1.4 | 1 | 1.7 | 0 | 0 | 0 | 0 | 2 | 4.3 | 3 | 0.8 |  |
| SLCO1B1 | *15\|*19 | P. Decreased | 2 | 0 | 2 | 0 | 0 | 0 | 0 | 0 | 0 | 0 | 0 | 0 | 0 | 0 |  |
| SLCO1B1 | *15\|*20 | Decreased | 171 | 1.3 | 159 | 1.2 | 2 | 3.3 | 3 | 5.4 | 0 | 0 | 0 | 0 | 7 | 1.8 |  |
| SLCO1B1 | *15\|*37 | Decreased | 242 | 1.8 | 222 | 1.7 | 4 | 6.7 | 1 | 1.8 | 0 | 0 | 8 | 17.4 | 7 | 1.8 |  |
| SLCO1B1 | *15\|*40 | P. Decreased | 1 | 0 | 1 | 0 | 0 | 0 | 0 | 0 | 0 | 0 | 0 | 0 | 0 | 0 |  |
| SLCO1B1 | *15\|*5 | Poor | 106 | 0.8 | 104 | 0.8 | 0 | 0 | 0 | 0 | 0 | 0 | 0 | 0 | 2 | 0.5 |  |
| SLCO1B1 | *19\|*20 | IND | 1 | 0 | 1 | 0 | 0 | 0 | 0 | 0 | 0 | 0 | 0 | 0 | 0 | 0 |  |
| SLCO1B1 | *19\|*37 | IND | 2 | 0 | 2 | 0 | 0 | 0 | 0 | 0 | 0 | 0 | 0 | 0 | 0 | 0 |  |
| SLCO1B1 | *1\|*14 | Normal | 2423 | 17.7 | 2360 | 18 | 2 | 3.3 | 5 | 8.9 | 0 | 0 | 5 | 10.9 | 51 | 13.4 |  |
| SLCO1B1 | *1\|*15 | Decreased | 1954 | 14.3 | 1895 | 14.4 | 1 | 1.7 | 8 | 14.3 | 0 | 0 | 6 | 13 | 44 | 11.5 |  |
| SLCO1B1 | *1\|*19 | IND | 19 | 0.1 | 18 | 0.1 | 0 | 0 | 0 | 0 | 0 | 0 | 0 | 0 | 1 | 0.3 |  |
| SLCO1B1 | *1\|*20 | Normal | 846 | 6.2 | 821 | 6.3 | 4 | 6.7 | 0 | 0 | 0 | 0 | 1 | 2.2 | 20 | 5.2 |  |
| SLCO1B1 | *1\|*26 | IND | 1 | 0 | 1 | 0 | 0 | 0 | 0 | 0 | 0 | 0 | 0 | 0 | 0 | 0 |  |
| SLCO1B1 | *1\|*27 | IND | 4 | 0 | 4 | 0 | 0 | 0 | 0 | 0 | 0 | 0 | 0 | 0 | 0 | 0 |  |
| SLCO1B1 | *1\|*30 | IND | 2 | 0 | 2 | 0 | 0 | 0 | 0 | 0 | 0 | 0 | 0 | 0 | 0 | 0 |  |
| SLCO1B1 | *1\|*31 | Decreased | 1 | 0 | 1 | 0 | 0 | 0 | 0 | 0 | 0 | 0 | 0 | 0 | 0 | 0 |  |
| SLCO1B1 | *1\|*37 | Normal | 963 | 7 | 891 | 6.8 | 18 | 30 | 10 | 17.9 | 4 | 50 | 10 | 21.7 | 30 | 7.9 |  |
| SLCO1B1 | *1\|*4 | IND | 1 | 0 | 1 | 0 | 0 | 0 | 0 | 0 | 0 | 0 | 0 | 0 | 0 | 0 |  |
| SLCO1B1 | *1\|*41 | IND | 1 | 0 | 1 | 0 | 0 | 0 | 0 | 0 | 0 | 0 | 0 | 0 | 0 | 0 |  |
| SLCO1B1 | *1\|*43 | IND | 1 | 0 | 0 | 0 | 0 | 0 | 1 | 1.8 | 0 | 0 | 0 | 0 | 0 | 0 |  |
| SLCO1B1 | *1\|*5 | Decreased | 533 | 3.9 | 504 | 3.8 | 0 | 0 | 3 | 5.4 | 0 | 0 | 0 | 0 | 26 | 6.8 |  |
| SLCO1B1 | *20\|*20 | Increased | 33 | 0.2 | 32 | 0.2 | 0 | 0 | 0 | 0 | 0 | 0 | 0 | 0 | 1 | 0.3 |  |
| SLCO1B1 | *20\|*37 | Normal | 84 | 0.6 | 74 | 0.6 | 2 | 3.3 | 3 | 5.4 | 0 | 0 | 1 | 2.2 | 4 | 1 |  |
| SLCO1B1 | *20\|*5 | Decreased | 53 | 0.4 | 51 | 0.4 | 0 | 0 | 1 | 1.8 | 0 | 0 | 0 | 0 | 1 | 0.3 |  |
| SLCO1B1 | *37\|*37 | Normal | 67 | 0.5 | 55 | 0.4 | 6 | 10 | 1 | 1.8 | 2 | 25 | 2 | 4.3 | 1 | 0.3 |  |
| SLCO1B1 | *37\|*40 | IND | 2 | 0 | 2 | 0 | 0 | 0 | 0 | 0 | 0 | 0 | 0 | 0 | 0 | 0 |  |
| SLCO1B1 | *37\|*41 | IND | 2 | 0 | 1 | 0 | 0 | 0 | 0 | 0 | 1 | 12.5 | 0 | 0 | 0 | 0 |  |
| SLCO1B1 | *37\|*5 | Decreased | 68 | 0.5 | 67 | 0.5 | 0 | 0 | 0 | 0 | 0 | 0 | 0 | 0 | 1 | 0.3 |  |
| SLCO1B1 | *5\|*5 | Poor | 14 | 0.1 | 13 | 0.1 | 0 | 0 | 0 | 0 | 0 | 0 | 0 | 0 | 1 | 0.3 |  |
| TPMT | *1\|*1 | NM | 12247 | 89.6 | 11752 | 89.6 | 58 | 96.7 | 48 | 85.7 | 6 | 75 | 40 | 87 | 343 | 90 |  |
| TPMT | *12\|*3A | IM | 1 | 0 | 1 | 0 | 0 | 0 | 0 | 0 | 0 | 0 | 0 | 0 | 0 | 0 |  |
| TPMT | *12\|*3C | IM | 1 | 0 | 1 | 0 | 0 | 0 | 0 | 0 | 0 | 0 | 0 | 0 | 0 | 0 |  |
| TPMT | *1\|*12 | IND | 12 | 0.1 | 11 | 0.1 | 0 | 0 | 0 | 0 | 0 | 0 | 0 | 0 | 1 | 0.3 |  |
| TPMT | *1\|*2 | IM | 62 | 0.5 | 61 | 0.5 | 0 | 0 | 0 | 0 | 0 | 0 | 0 | 0 | 1 | 0.3 |  |
| TPMT | *1\|*21 | IND | 1 | 0 | 1 | 0 | 0 | 0 | 0 | 0 | 0 | 0 | 0 | 0 | 0 | 0 |  |
| TPMT | *1\|*24 | IND | 1 | 0 | 1 | 0 | 0 | 0 | 0 | 0 | 0 | 0 | 0 | 0 | 0 | 0 |  |
| TPMT | *1\|*37 | IND | 1 | 0 | 1 | 0 | 0 | 0 | 0 | 0 | 0 | 0 | 0 | 0 | 0 | 0 |  |
| TPMT | *1\|*3A | IM | 1128 | 8.3 | 1089 | 8.3 | 1 | 1.7 | 5 | 8.9 | 0 | 0 | 3 | 6.5 | 30 | 7.9 |  |
| TPMT | *1\|*3C | IM | 151 | 1.1 | 143 | 1.1 | 1 | 1.7 | 1 | 1.8 | 1 | 12.5 | 1 | 2.2 | 4 | 1 |  |
| TPMT | *1\|*43 | IND | 1 | 0 | 1 | 0 | 0 | 0 | 0 | 0 | 0 | 0 | 0 | 0 | 0 | 0 |  |
| TPMT | *1\|*8 | IND | 7 | 0.1 | 5 | 0 | 0 | 0 | 1 | 1.8 | 1 | 12.5 | 0 | 0 | 0 | 0 |  |
| TPMT | *1\|*9 | IND | 26 | 0.2 | 24 | 0.2 | 0 | 0 | 0 | 0 | 0 | 0 | 1 | 2.2 | 1 | 0.3 |  |
| TPMT | *2\|*3A | PM | 1 | 0 | 1 | 0 | 0 | 0 | 0 | 0 | 0 | 0 | 0 | 0 | 0 | 0 |  |
| TPMT | *3A\|*3A | PM | 23 | 0.2 | 22 | 0.2 | 0 | 0 | 0 | 0 | 0 | 0 | 0 | 0 | 1 | 0.3 |  |
| TPMT | *3A\|*3C | PM | 6 | 0 | 4 | 0 | 0 | 0 | 1 | 1.8 | 0 | 0 | 1 | 2.2 | 0 | 0 |  |
| TPMT | *3A\|*9 | IM | 1 | 0 | 1 | 0 | 0 | 0 | 0 | 0 | 0 | 0 | 0 | 0 | 0 | 0 |  |
| VKORC1 | *1\|*1 | Normal | 5320 | 38.9 | 5101 | 38.9 | 36 | 60 | 14 | 25 | 3 | 37.5 | 15 | 32.6 | 151 | 39.6 |  |
| VKORC1 | *1\|*2 | Intermediate | 6444 | 47.1 | 6191 | 47.2 | 22 | 36.7 | 27 | 48.2 | 2 | 25 | 26 | 56.5 | 176 | 46.2 |  |
| VKORC1 | *2\|*2 | Low | 1906 | 13.9 | 1827 | 13.9 | 2 | 3.3 | 15 | 26.8 | 3 | 37.5 | 5 | 10.9 | 54 | 14.2 |  |
| AFR, African; AMR, American; EUR, European; SAS, Central/South Asian; CYP2D6 copy number and structural variants (e.g., *xN, *5, *13, *36, *68) were not detectable, which could impact the accuracy of the CYP2D6 diplotype frequencies. VKORC1 *2 = rs9923231 A allele; IM, intermediate metabolizer; NM, normal metabolizer; PM, poor metabolizer; RM, rapid metabolizer; UM, ultrarapid metabolizer, P. Decreased = Possibly Decreased | | | | | | | | | | | | | | | | |  |
|  |  |  |  |  |  |  |  |  |  |  |  |  |  |  |  |  |  |

| **Supplementary Table S4.** Genotype-predicted phenotype frequencies. | | | | | | | | | | | | | | | |
| --- | --- | --- | --- | --- | --- | --- | --- | --- | --- | --- | --- | --- | --- | --- | --- |
|  |  | Full Sample (13,670) | | EUR (13,119) | | SAS (60) | | AMR (56) | | AFR (8) | | Mixed (46) | | Unknown (381) | |
| Gene | Phenotype | N | % | N | % | N | % | N | % | N | % | N | % | N | % |
| CYP2B6 | Indeterminate | 368 | 2.7 | 354 | 2.7 | 0 | 0 | 0 | 0 | 0 | 0 | 0 | 0 | 14 | 3.7 |
| CYP2B6 | Normal | 6983 | 51.1 | 6718 | 51.2 | 20 | 33.3 | 28 | 50 | 4 | 50 | 23 | 50 | 190 | 49.9 |
| CYP2B6 | Intermediate | 4987 | 36.5 | 4770 | 36.4 | 28 | 46.7 | 25 | 44.6 | 2 | 25 | 19 | 41.3 | 143 | 37.5 |
| CYP2B6 | Poor | 835 | 6.1 | 794 | 6.1 | 10 | 16.7 | 3 | 5.4 | 2 | 25 | 2 | 4.3 | 24 | 6.3 |
| CYP2B6 | Rapid | 488 | 3.6 | 475 | 3.6 | 2 | 3.3 | 0 | 0 | 0 | 0 | 1 | 2.2 | 10 | 2.6 |
| CYP2B6 | Ultrarapid | 8 | 0.1 | 7 | 0.1 | 0 | 0 | 0 | 0 | 0 | 0 | 1 | 2.2 | 0 | 0 |
| CYP2B6 | Not available | 1 | 0 | 1 | 0 | 0 | 0 | 0 | 0 | 0 | 0 | 0 | 0 | 0 | 0 |
| CYP2C19 | Normal | 5515 | 40.3 | 5309 | 40.5 | 10 | 16.7 | 28 | 50 | 3 | 37.5 | 14 | 30.4 | 151 | 39.6 |
| CYP2C19 | Intermediate | 3544 | 25.9 | 3373 | 25.7 | 27 | 45 | 9 | 16.1 | 4 | 50 | 22 | 47.8 | 109 | 28.6 |
| CYP2C19 | Poor | 328 | 2.4 | 301 | 2.3 | 12 | 20 | 1 | 1.8 | 0 | 0 | 1 | 2.2 | 13 | 3.4 |
| CYP2C19 | Rapid | 3622 | 26.5 | 3490 | 26.6 | 11 | 18.3 | 16 | 28.6 | 1 | 12.5 | 8 | 17.4 | 96 | 25.2 |
| CYP2C19 | Ultrarapid | 656 | 4.8 | 641 | 4.9 | 0 | 0 | 2 | 3.6 | 0 | 0 | 1 | 2.2 | 12 | 3.1 |
| CYP2C19 | Indeterminate | 5 | 0 | 5 | 0 | 0 | 0 | 0 | 0 | 0 | 0 | 0 | 0 | 0 | 0 |
| CYP2C9 | Normal | 8651 | 63.3 | 8265 | 63 | 49 | 81.7 | 44 | 78.6 | 6 | 75 | 33 | 71.7 | 254 | 66.7 |
| CYP2C9 | Intermediate | 3078 | 22.5 | 2970 | 22.6 | 3 | 5 | 9 | 16.1 | 0 | 0 | 10 | 21.7 | 86 | 22.6 |
| CYP2C9 | Intermediate (AS=1) | 1684 | 12.3 | 1632 | 12.4 | 8 | 13.3 | 3 | 5.4 | 2 | 25 | 3 | 6.5 | 36 | 9.4 |
| CYP2C9 | Poor | 253 | 1.9 | 248 | 1.9 | 0 | 0 | 0 | 0 | 0 | 0 | 0 | 0 | 5 | 1.3 |
| CYP2C9 | Not available | 4 | 0 | 4 | 0 | 0 | 0 | 0 | 0 | 0 | 0 | 0 | 0 | 0 | 0 |
| CYP2D6 | Normal | 7765 | 56.8 | 7434 | 56.7 | 44 | 73.3 | 43 | 76.8 | 5 | 62.5 | 30 | 65.2 | 209 | 54.9 |
| CYP2D6 | Intermediate | 4861 | 35.6 | 4678 | 35.7 | 13 | 21.7 | 9 | 16.1 | 2 | 25 | 13 | 28.3 | 146 | 38.3 |
| CYP2D6 | Poor | 789 | 5.8 | 765 | 5.8 | 3 | 5 | 0 | 0 | 1 | 12.5 | 2 | 4.3 | 18 | 4.7 |
| CYP2D6 | Indeterminate | 242 | 1.8 | 230 | 1.8 | 0 | 0 | 4 | 7.1 | 0 | 0 | 1 | 2.2 | 7 | 1.8 |
| CYP2D6 | Not available | 13 | 0.1 | 12 | 0.1 | 0 | 0 | 0 | 0 | 0 | 0 | 0 | 0 | 1 | 0.3 |
| CYP3A5 | Normal | 60 | 0.4 | 49 | 0.4 | 4 | 6.7 | 5 | 8.9 | 1 | 12.5 | 0 | 0 | 1 | 0.3 |
| CYP3A5 | Intermediate | 1629 | 11.9 | 1537 | 11.7 | 24 | 40 | 17 | 30.4 | 2 | 25 | 12 | 26.1 | 37 | 9.7 |
| CYP3A5 | Poor | 11981 | 87.6 | 11533 | 87.9 | 32 | 53.3 | 34 | 60.7 | 5 | 62.5 | 34 | 73.9 | 343 | 90 |
| DPYD | Normal Metabolizer | 12775 | 93.5 | 12247 | 93.4 | 57 | 95 | 56 | 100 | 7 | 87.5 | 45 | 97.8 | 363 | 95.3 |
| DPYD | Intermediate Metabolizer | 889 | 6.5 | 866 | 6.6 | 3 | 5 | 0 | 0 | 1 | 12.5 | 1 | 2.2 | 18 | 4.7 |
| DPYD | Poor Metabolizer | 6 | 0 | 6 | 0 | 0 | 0 | 0 | 0 | 0 | 0 | 0 | 0 | 0 | 0 |
| NUDT15 | Normal | 13358 | 97.7 | 12838 | 97.9 | 49 | 81.7 | 47 | 83.9 | 8 | 100 | 42 | 91.3 | 374 | 98.2 |
| NUDT15 | Intermediate | 240 | 1.8 | 212 | 1.6 | 11 | 18.3 | 9 | 16.1 | 0 | 0 | 4 | 8.7 | 4 | 1 |
| NUDT15 | Not available | 14 | 0.1 | 13 | 0.1 | 0 | 0 | 0 | 0 | 0 | 0 | 0 | 0 | 1 | 0.3 |
| NUDT15 | Indeterminate | 58 | 0.4 | 56 | 0.4 | 0 | 0 | 0 | 0 | 0 | 0 | 0 | 0 | 2 | 0.5 |
| SLCO1B1 | Normal | 9065 | 66.3 | 8688 | 66.2 | 50 | 83.3 | 37 | 66.1 | 7 | 87.5 | 27 | 58.7 | 256 | 67.2 |
| SLCO1B1 | Decreased | 3668 | 26.8 | 3526 | 26.9 | 7 | 11.7 | 16 | 28.6 | 0 | 0 | 15 | 32.6 | 104 | 27.3 |
| SLCO1B1 | Possible Decreased | 3 | 0 | 3 | 0 | 0 | 0 | 0 | 0 | 0 | 0 | 0 | 0 | 0 | 0 |
| SLCO1B1 | Increased | 579 | 4.2 | 559 | 4.3 | 2 | 3.3 | 2 | 3.6 | 0 | 0 | 2 | 4.3 | 14 | 3.7 |
| SLCO1B1 | Poor | 308 | 2.3 | 299 | 2.3 | 1 | 1.7 | 0 | 0 | 0 | 0 | 2 | 4.3 | 6 | 1.6 |
| SLCO1B1 | Indeterminate | 47 | 0.3 | 44 | 0.3 | 0 | 0 | 1 | 1.8 | 1 | 12.5 | 0 | 0 | 1 | 0.3 |
| TPMT | Normal | 12247 | 89.6 | 11752 | 89.6 | 58 | 96.7 | 48 | 85.7 | 6 | 75 | 40 | 87 | 343 | 90 |
| TPMT | Intermediate | 1344 | 9.8 | 1296 | 9.9 | 2 | 3.3 | 6 | 10.7 | 1 | 12.5 | 4 | 8.7 | 35 | 9.2 |
| TPMT | Poor | 30 | 0.2 | 27 | 0.2 | 0 | 0 | 1 | 1.8 | 0 | 0 | 1 | 2.2 | 1 | 0.3 |
| TPMT | Indeterminate | 48 | 0.4 | 43 | 0.3 | 0 | 0 | 1 | 1.8 | 1 | 12.5 | 1 | 2.2 | 2 | 0.5 |
| TPMT | Not available | 1 | 0 | 1 | 0 | 0 | 0 | 0 | 0 | 0 | 0 | 0 | 0 | 0 | 0 |
| VKORC1 | Normal | 5320 | 38.9 | 5101 | 38.9 | 36 | 60 | 14 | 25 | 3 | 37.5 | 15 | 32.6 | 151 | 39.6 |
| VKORC1 | Intermediate | 6444 | 47.1 | 6191 | 47.2 | 22 | 36.7 | 27 | 48.2 | 2 | 25 | 26 | 56.5 | 176 | 46.2 |
| VKORC1 | Low | 1906 | 13.9 | 1827 | 13.9 | 2 | 3.3 | 15 | 26.8 | 3 | 37.5 | 5 | 10.9 | 54 | 14.2 |
| AFR, African; AMR, American; EUR, European; SAS, Central/South Asian; CYP2D6 copy number and structural variants (e.g., *xN, *5, *13, *36, *68) were not detectable, which could impact the accuracy of the CYP2D6 phenotype frequencies. VKORC1 Normal (rs9923231 GG or *1/*1), Intermediate (rs9923231 AG or *1/*2), Low (rs9923231 AA or *2/*2) | | | | | | | | | | | | | | | |

**Supplementary Table S5.** Cautionary medication use and actionability among 13.670 older adults

|  |  | **Used Medication** | |  | **Actionable** | |
| --- | --- | --- | --- | --- | --- | --- |
| **Cautionary medication** | **Gene(s) with actionable recommendations** | **N** | **%** |  | **N** | **%** |
| atorvastatin | SLCO1B1 | 2264 | 16.7% |  | 641 | 28.3% |
| rosuvastatin | SLCO1B1 | 1811 | 13.4% |  | 534 | 29.5% |
| meloxicam | CYP2C9 | 1414 | 10.4% |  | 193 | 13.6% |
| pantoprazole | CYP2C19 | 1360 | 10.0% |  | 54 | 4.0% |
| simvastatin | SLCO1B1 | 1339 | 9.9% |  | 406 | 30.3% |
| celecoxib | CYP2C9 | 893 | 6.6% |  | 143 | 16.0% |
| omeprazole | CYP2C19 | 759 | 5.6% |  | 38 | 5.0% |
| metoprolol | CYP2D6 | 755 | 5.6% |  | 300 | 39.7% |
| codeine | CYP2D6 | 642 | 4.7% |  | 282 | 43.9% |
| amitriptyline | CYP2C19, CYP2D6 | 603 | 4.5% |  | 376 | 62.4% |
| tramadol | CYP2D6 | 412 | 3.0% |  | 168 | 40.8% |
| sertraline | CYP2B6, CYP2C19 | 393 | 2.9% |  | 227 | 57.8% |
| ibuprofen | CYP2C9 | 382 | 2.8% |  | 51 | 13.4% |
| escitalopram | CYP2C19 | 282 | 2.1% |  | 101 | 35.8% |
| warfarin | CYP2C9, VKORC1 | 269 | 2.0% |  | 202 | 75.1% |
| clopidogrel | CYP2C19 | 254 | 1.9% |  | 63 | 24.8% |
| pravastatin | SLCO1B1 | 250 | 1.8% |  | 75 | 30.0% |
| citalopram | CYP2C19 | 200 | 1.5% |  | 69 | 34.5% |
| venlafaxine | CYP2D6 | 183 | 1.4% |  | 70 | 38.3% |
| paroxetine | CYP2D6 | 157 | 1.2% |  | 64 | 40.8% |
| lansoprazole | CYP2C19 | 138 | 1.0% |  | 85 | 61.6% |
| fluorouracil | DPYD | 119 | 0.9% |  | 5 | 4.2% |
| tamoxifen | CYP2D6 | 89 | 0.7% |  | 41 | 46.1% |
| piroxicam | CYP2C9 | 75 | 0.6% |  | 5 | 6.7% |
| flecainide | CYP2D6 | 52 | 0.4% |  | 21 | 40.4% |
| doxepin | CYP2C19, CYP2D6 | 49 | 0.4% |  | 35 | 71.4% |
| fluvoxamine | CYP2D6 | 36 | 0.3% |  | 1 | 2.8% |
| hydrocodone | CYP2D6 | 34 | 0.3% |  | 13 | 38.2% |
| ondansetron | CYP2D6 | 33 | 0.2% |  | 0 | 0.0% |
| phenytoin | CYP2C9 | 32 | 0.2% |  | 10 | 31.3% |
| lovastatin | SLCO1B1 | 24 | 0.2% |  | 8 | 33.3% |
| azathioprine | NUDT15, TPMT | 22 | 0.2% |  | 3 | 13.6% |
| risperidone | CYP2D6 | 19 | 0.1% |  | 0 | 0.0% |
| imipramine | CYP2C19, CYP2D6 | 17 | 0.1% |  | 7 | 41.2% |
| fluvastatin | CYP2C9, SLCO1B1 | 16 | 0.1% |  | 9 | 56.3% |
| capecitabine | DPYD | 16 | 0.1% |  | 1 | 6.3% |
| haloperidol | CYP2D6 | 12 | 0.1% |  | 2 | 16.7% |
| clomipramine | CYP2C19, CYP2D6 | 11 | 0.1% |  | 7 | 63.6% |
| tacrolimus | CYP3A5 | 5 | 0.0% |  | 1 | 20.0% |
| mercaptopurine | NUDT15, TPMT | 4 | 0.0% |  | 0 | 0.0% |
| pitavastatin | SLCO1B1 | 2 | 0.0% |  | 0 | 0.0% |
| voriconazole | CYP2C19 | 2 | 0.0% |  | 1 | 50.0% |
| desipramine | CYP2D6 | 2 | 0.0% |  | 0 | 0.0% |
| propafenone | CYP2D6 | 1 | 0.0% |  | 0 | 0.0% |
| trimipramine | CYP2C19, CYP2D6 | 1 | 0.0% |  | 1 | 100.0% |
| irinotecan | UGT1A1 | 1 | 0.0% |  | 0 | 0.0% |
| efavirenz | CYP2B6 | 1 | 0.0% |  | 1 | 100.0% |
| atomoxetine | CYP2D6 | 1 | 0.0% |  | 0 | 0.0% |

The proportion of participants with a CYP2D6 actionable genotype could be imprecise because copy number and structural variants (e.g., *xN, *5, *13, *36, *68) were not detectable.

**Supplementary Table S6.** Inhibitor and inducer medication use among 13,670 older adults

| **Gene** | **Inhibitors** | **%** |  | **Inducers** | **%** |
| --- | --- | --- | --- | --- | --- |
| CYP3A5 | amlodipine | 22.86 |  | betamethasone | 7.10 |
|  | clarithromycin | 0.60 |  | carbamazepine | 0.78 |
|  | diltiazem | 2.88 |  | clobazam | 0.02 |
|  | erythromycin | 0.33 |  | dabrafenib | 0.02 |
|  | fluconazole | 0.11 |  | dexamethasone | 1.44 |
|  | imatinib | 0.02 |  | efavirenz | 0.01 |
|  | itraconazole | 0.02 |  | enzalutamide | 0.15 |
|  | nefazodone | 0.01 |  | methylprednisolone | 2.18 |
|  | verapamil | 2.70 |  | modafinil | 0.02 |
|  | voriconazole | 0.02 |  | nevirapine | 0.01 |
|  |  | |  | oxcarbazepine | 0.01 |
|  |  |  |  | phenobarbital | 0.05 |
|  |  |  |  | phenytoin | 0.36 |
|  |  |  |  | pioglitazone | 0.40 |
|  |  |  |  | prednisolone | 1.49 |
|  |  |  |  | prednisone | 10.47 |
| CYP2B6 | voriconazole | 0.02 |  | carbamazepine | 0.78 |
|  |  |  |  | efavirenz | 0.01 |
|  |  |  |  | nevirapine | 0.01 |
|  |  |  |  | phenytoin | 0.36 |
| CYP2D6 | abiraterone | 0.08 |  |  | |
|  | bupropion | 0.16 |  |  |  |
|  | cinacalcet | 0.01 |  |  |  |
|  | clobazam | 0.02 |  |  |  |
|  | doxepin | 0.56 |  |  |  |
|  | duloxetine | 1.19 |  |  |  |
|  | fluoxetine | 1.17 |  |  |  |
|  | moclobemide | 0.22 |  |  |  |
|  | paroxetine | 1.78 |  |  |  |
|  | terbinafine | 0.68 |  |  |  |
| CYP2C19 | esomeprazole | 28.71 |  | efavirenz | 0.01 |
|  | fluconazole | 0.11 |  | enzalutamide | 0.15 |
|  | fluoxetine | 1.17 |  | prednisone | 10.47 |
|  | fluvoxamine | 0.41 |  | rifampicin | 0.12 |
|  | omeprazole | 8.61 |  |  |  |
|  | voriconazole | 0.02 |  |  |  |
| CYP2C9 | amiodarone | 0.89 |  | carbamazepine | 0.78 |
|  | fluconazole | 0.11 |  | dabrafenib | 0.02 |
|  | metronidazole | 1.33 |  | enzalutamide | 0.15 |
|  |  | |  | nevirapine | 0.01 |
|  |  |  |  | phenobarbital | 0.05 |

Inhibitors and inducers according to the Drug Interactions Flockhart Table (<https://drug-interactions.medicine.iu.edu/MainTable.aspx)>.
